# Competing Modes of Hydrogen Activation in Singlet Pyridinylidenes: π-Approach *vs* σ*-Approach Reaction Pathways

**DOI:** 10.1021/acs.jpca.5c04771

**Published:** 2025-09-01

**Authors:** Gurli A. Schuster, Virinder Bhagat, J. Philipp Wagner

**Affiliations:** † Institut für Organische Chemie, 9168 Eberhard Karls Universität Tübingen, Auf der Morgenstelle 18, Tübingen 72076, Germany; ‡ Institut für Organische und Analytische Chemie, Universität Bremen, Leobener Straße 7, Bremen 28359, Germany

## Abstract

Carbenes are promising reagents for the transition metal-free activation of molecular hydrogen. Depending on their multiplicity and electron configuration, carbenes can access different hydrogenation reaction mechanisms, with singlet carbenes usually leading to geminal hydrogenation products *via* a π-approach trajectory. Our group has recently prepared 1-iodopyridine-2-ylidene, **1-I**, introducing a new class of singlet *N*-heterocyclic carbenes featuring σ/σ* instead of the usually encountered σ/π frontier orbitals. Carbene **1-I** reacts with H_2_ *via* a unique sideways σ*-approach, leading to pyridinium iodide formation with N−I bond cleavage. This study investigates how nitrogen substituents with varying bond strengths (I < Br < Cl < OCF_3_ < OMe < NMe_2_ < F < Me) influence the electronic structure of the carbene and its preference for σ*- or π-approach hydrogenation. Using high-accuracy DLPNO−CCSD­(T) and NEVPT2 computations, we find that I-, Br-, Cl-, and OCF_3_-substituted pyridinylidenes adopt a σ^2^σ^*0^ configuration and favor σ*-approach hydrogenation. In contrast, OMe-, NMe_2_-, and Me-substituted carbenes exhibit a σ^2^π^0^ configuration and lower barriers for the π-approach hydrogenation. Interestingly, the fluorine-substituted carbene assumes a σ^2^π^0^ electron configuration yet still preferentially undergoes σ*-approach hydrogenation.

## Graphical abstract

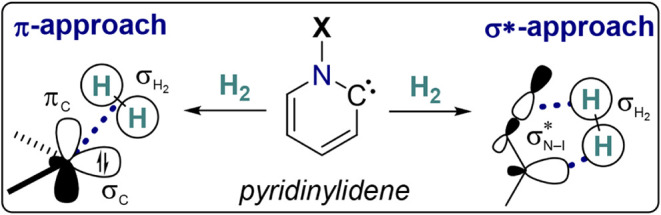

## 1 Introduction

The insertion reaction of carbenes with molecular hydrogen (H_2_) has been a topic of long-standing mechanistic interest.^1–8^ Beyond fundamental reactivity, this process also holds promise for the transition metal-free activation of H_2_.^9,10^ However, breaking the strong dihydrogen bond is inherently challenging due to its high bond dissociation energy (*BDE*_H−H_ = 104.2 kcal mol^−1^)^11^ and the absence of polarity, which limits straightforward modes of attack. Traditionally, transition metal complexes are employed to bind and activate H_2_ through the interplay of filled and vacant d-orbitals at the metal center.^12–14^ In this regard, Bertrand recognized that singlet carbenes exhibit an electronic structure analogous to transition metals, possessing a lone pair of electrons in a σ orbital and an orthogonal vacant π orbital (σ^2^π^0^-singlet, Figure 1A).^15^ This electronic configuration suggests that certain carbenes could activate H_2_ in a manner similar to transition metals. Supporting this hypothesis, Bertrand’s group demonstrated that stable nucleophilic (alkyl)­(amino)­carbenes, such as **I**, undergo insertion into the H−H bond near room temperature (Figure 1A).^15^ Furthermore, more electrophilic singlet carbenes have been shown to react with H_2_ even at cryogenic temperatures.^16,17^

**Figure 1:**
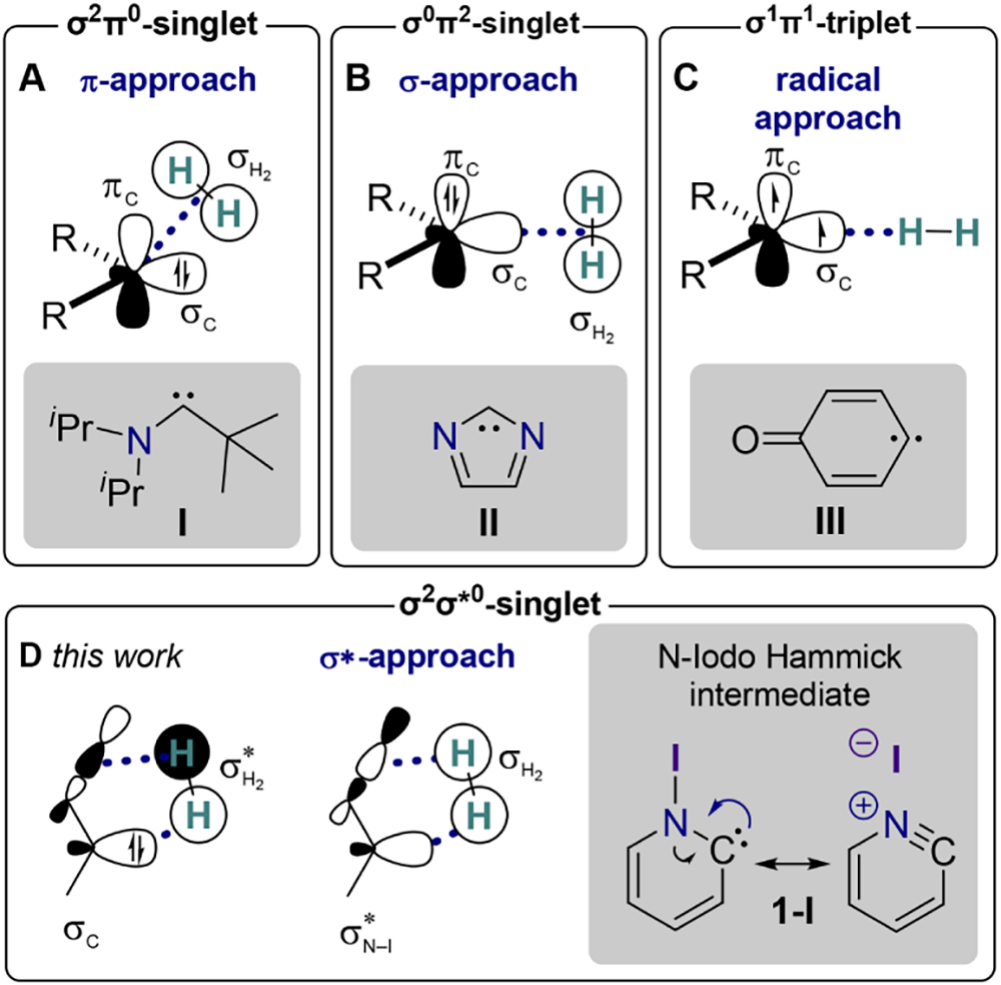
Carbenes can assume different electronic ground states depending on the occupation of a σ and a π frontier orbital including (A) the σ^2^π^0^-singlet, (B) the σ^0^π^2^-singlet, and (C) the σ^1^π^1^-triplet. (D) The N-iodo Hammick intermediate, **1-I**, features a σ^2^σ*^0^-singlet ground state due to a resonance of the lone pair of the carbene with the N−I bond. Depending on the electronic ground state, the reaction with molecular hydrogen traverses a different approach trajectory. Examples are highlighted in gray.

Singlet carbenes can adopt different electronic configurations, with the σ^2^π^0^ state being the more commonly observed ground state.^18^ However, in certain cases, a formally doubly excited σ^0^π^2^ configuration can also be energetically accessible, as exemplified by carbene **II** (Figure 1B).^19–22^ These differences in electronic structure profoundly influence their reactivity with molecular hydrogen (H_2_). Conventional σ^2^π^0^ carbenes undergo hydrogenation *via* a non-least-motion *π-approach*, dictated by the constraints of orbital symmetry conservation (Figure 1A).^2,3,23^ This trajectory ensures optimal overlap between the H_2_ σ orbital and the vacant carbene π orbital, as well as between the filled carbene σ orbital and the H_2_ σ* orbital, leading to bond activation. Conversely, σ^0^π^2^ carbenes can react through a least-motion *σ-approach* reaction pathway, allowing direct addition of H_2_ without significant structural reorganization while still maximizing favorable frontier orbital interactions (Figure 1B).^7,8^

Yet another reaction mechanism emerges in triplet carbenes, which adopt a σ^1^π^1^ electronic configuration (Figure 1C). These species behave as diradicals, reacting with H_2_ *via* hydrogen atom abstraction,^24^ generating a triplet radical pair consisting of a monohydrogenated carbene and a free hydrogen atom.^25^ The radical pair can subsequently undergo intersystem crossing to the singlet state, facilitating collapse into the geminal dihydrogenation product.^26^ Notably, numerous triplet carbenes have been observed to undergo hydrogenation even under cold matrix isolation conditions, suggesting that the reaction is driven by quantum mechanical tunneling (QMT).^25–30^

In addition to the well-established classes of carbenes characterized by the occupation of σ and π frontier orbitals, our laboratory has recently identified a new class of N-heterocyclic carbenes with a fundamentally different electronic structure.^31^ In these carbenes, the vacant frontier orbital is not associated with the π system but rather with the σ* orbital of the bond between nitrogen and its substituent (Figure 1D). In particular, we have spectroscopically characterized the N-iodo Hammick intermediate, **1-I**, a pyridine-2-ylidene featuring an N−I bond, generated by UV irradiation of 2-iodopyridine in a low temperature neon matrix (Scheme 1). Despite being a singlet carbene, **1-I** exhibits a rather large bond angle at the carbene center exceeding 120°, which facilitates a resonance interaction between the carbene σ and the N−I bond’s σ* orbital. This leads to a strong mixing of both orbitals (*cf.* Figure 1D) and pronounced deviation from integral electron occupation numbers in a complete active space self-consistent field (CASSCF) treatment of the molecule.

**Scheme 1:**
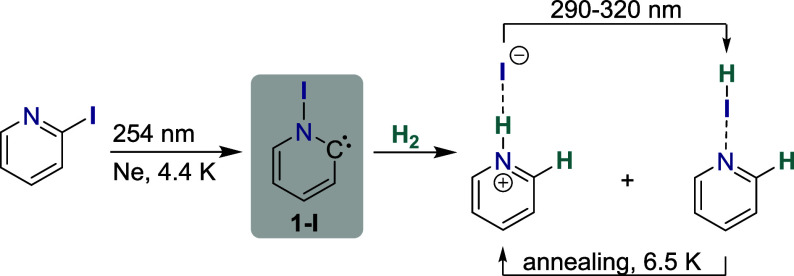
Synthesis and Hydrogenation Reaction of the N-Iodo Hammick Intermediate **1-I** in Matrix Isolation

The altered electronic structure of σ^2^σ*^0^ carbenes introduces a new hydrogenation pathway through an in-plane addition of dihydrogen *via* the σ* approach (Figure 1D).^31^ In this mechanism, the carbene σ orbital interacts with dihydrogen’s σ* orbital, while a simultaneous electron back-donation occurs from the H_2_ σ orbital into the σ* orbital of the carbene, facilitating bond activation. Experimentally, it could be demonstrated that pyridinylidene **1-I** reacts with H_2_ in neon matrix isolation, producing pyridinium iodide and a halogen-bound complex of pyridine with hydrogen iodide (Scheme 1). The latter species can be further accumulated photochemically (λ = 290−320 nm) and reversibly converts back upon annealing of the matrix at 6.5 K. Notably, strong experimental evidence supports quantum mechanical tunneling (QMT) as the key driving factor behind the H_2_ fission reaction. This conclusion is confirmed by the observed kinetic isotope effect, as H_2_ reacts readily, whereas D_2_ remains unreactive under identical conditions. Most interestingly, instanton theory computations predict a change of mechanism from a concerted to a hydrogen atom abstraction mechanism at the lowest experimental temperatures through *tunneling control*.^31,32^

**T**he preference for an in-plane (σ* approach) hydrogenation in pyridinylidene **1-I** contrasts sharply with the previously reported π-approach hydrogenation of a pyridinylidene featuring a bulky aryl substituent at nitrogen. In the latter case, hydrogenation follows an out of plane trajectory as depicted in Figure 1A, yielding a geminal dihydrogenation product.^33–35^ These divergent reaction pathways arise from competing electronic structures in pyridinylidenes, governed by the occupation of σ/π *versus* σ/σ* frontier orbitals. Crucially, this orbital preference is strongly influenced by the binding strength of the substituent and the associated energetic availability of the N−X bond’s σ* orbital.^31^ To further explore this relationship, we set out to conduct a computational study investigating the substituent-dependent electronic structures of pyridinylidenes and assess their impact on the preference for σ*- *versus* π-approach hydrogenation mechanisms, as seen in Scheme 2.

**Scheme 2:**
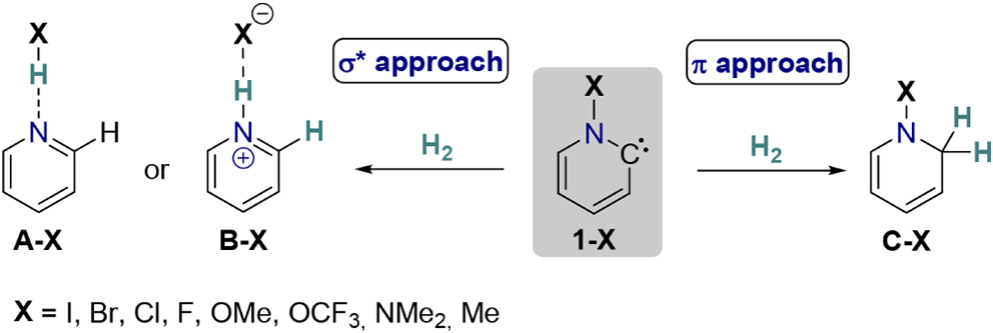
Hydrogenation Reactions of Pyridinylidenes *via* the Competing σ* and π Approach Reaction Trajectories Depending on the Substituent on the Nitrogen Atom

## 2 Computational Methods

The geometric structures of various substituted pyridine-2-ylidenes, along with their corresponding hydrogenation transition states (TSs) and products, were optimized at the B3LYP-D3/def2-TZVPP level of theory using the Gaussian16 electronic structure code.^36–41^ Vibrational frequency computations confirmed the nature of the optimized stationary points and provided zero-point vibrational energy corrections. Additionally, intrinsic reaction coordinate (IRC) analyses were performed for all transition states to verify their correspondence to the desired reaction pathway.^42^ We generally avoided the use of broken-symmetry solutions unless stated otherwise, as subsequent multireference calculations confirmed that restricted Kohn−Sham solutions were energetically preferred for all carbenes.

Given the complex electronic structure of substituted pyridinylidenes, we pursued two strategies to improve the accuracy of the electronic energies. First, we performed n-electron valence state perturbation theory (NEVPT2) single-point energy calculations without the frozen-core approximation and a def2-TZVPP basis set for all hydrogenation transition states.^43^ These computations were based on a CASSCF-(12,11) wave function,^44,45^ incorporating the σ lone pair and π orbitals of the carbene in the ring, the bonding σ and antibonding σ* orbitals of the nitrogen substituent, and the bonding σ and antibonding σ* orbital of dihydrogen in the active space.^31^ Second, we refined the electronic energies by performing single-point calculations using domain-based local pair natural orbital (DLPNO) coupled-cluster theory with single, double, and perturbative triple excitations [DLPNO−CCSD­(T)], in combination with a def2-QZVPP basis set and TightPNO settings.^46–48^ While pyridinylidenes with σ/σ* frontier orbitals exhibit elevated *T*_1_ diagnostic values, they only slightly exceed the recommended threshold of 0.02 (see Table S1). Thus, we consider single-reference coupled-cluster theory sufficiently reliable to accurately capture the key trends in electronic structure relevant to this study. This approach also eliminates the need for extending the active space when computing singlet−triplet energy gaps (Δ*E*_S-T_) and N−X bond dissociation energies (*BDE*_N−X_). All high-level single point energies were obtained utilizing the ORCA 5 quantum chemistry package.^49^

## 3 Results and Discussion

### 3.1 Carbene Structures and Electron Configurations

To explore the full range of electronic structures in pyridinylidenes, from σ^2^σ*^0^ to σ^2^π^0^ configurations, we selected eight substituents (X) with varying bond energies to nitrogen, ranging from iodine with a lower bond dissociation energy to the methyl group with a higher bond dissociation energy (Scheme 2). We included the halogens Br, Cl, and F, as well as the methoxy (OMe), trifluoromethoxy (OCF_3_), and dimethylamino (NMe_2_) groups, extending our previous analysis.^31^ The optimized structures of all eight singlet carbenes, their Kohn−Sham frontier orbitals, along with the computed N−X bond dissociation energies (*BDE*_N−X_) and singlet−triplet energy gaps (Δ*E*_S-T_) are collected in Figure 2. Since the electronic structure of the carbene appears to depend strongly on the substituent’s bond dissociation energy, i.e., the dissociation into X^•^ and 2-pyridyl radical, the pyridinylidenes are ordered accordingly. The binding energies, computed at the DLPNO−CCSD­(T)/def2-TZVPP//B3LYP-D3/def2-TZVPP level of theory, range from 9.4 kcal mol^−1^ for carbene **1-I** to 47.6 kcal mol^−1^ for **1-Me**. The other carbenes assume intermediate values of 14.0 kcal mol^−1^ (**1-Br**), 17.8 kcal mol^−1^ (**1-Cl**), 22.4 kcal mol^−1^ (**1-OCF**_**3**_), 23.2 kcal mol^−1^ (**1-OCH**_**3**_), 29.4 kcal mol^−1^ (**1-NMe**_**2**_), and 34.6 kcal mol^−1^ (**1-F**). This ordering by bond dissociation energy is used consistently throughout this work.

**Figure 2:**
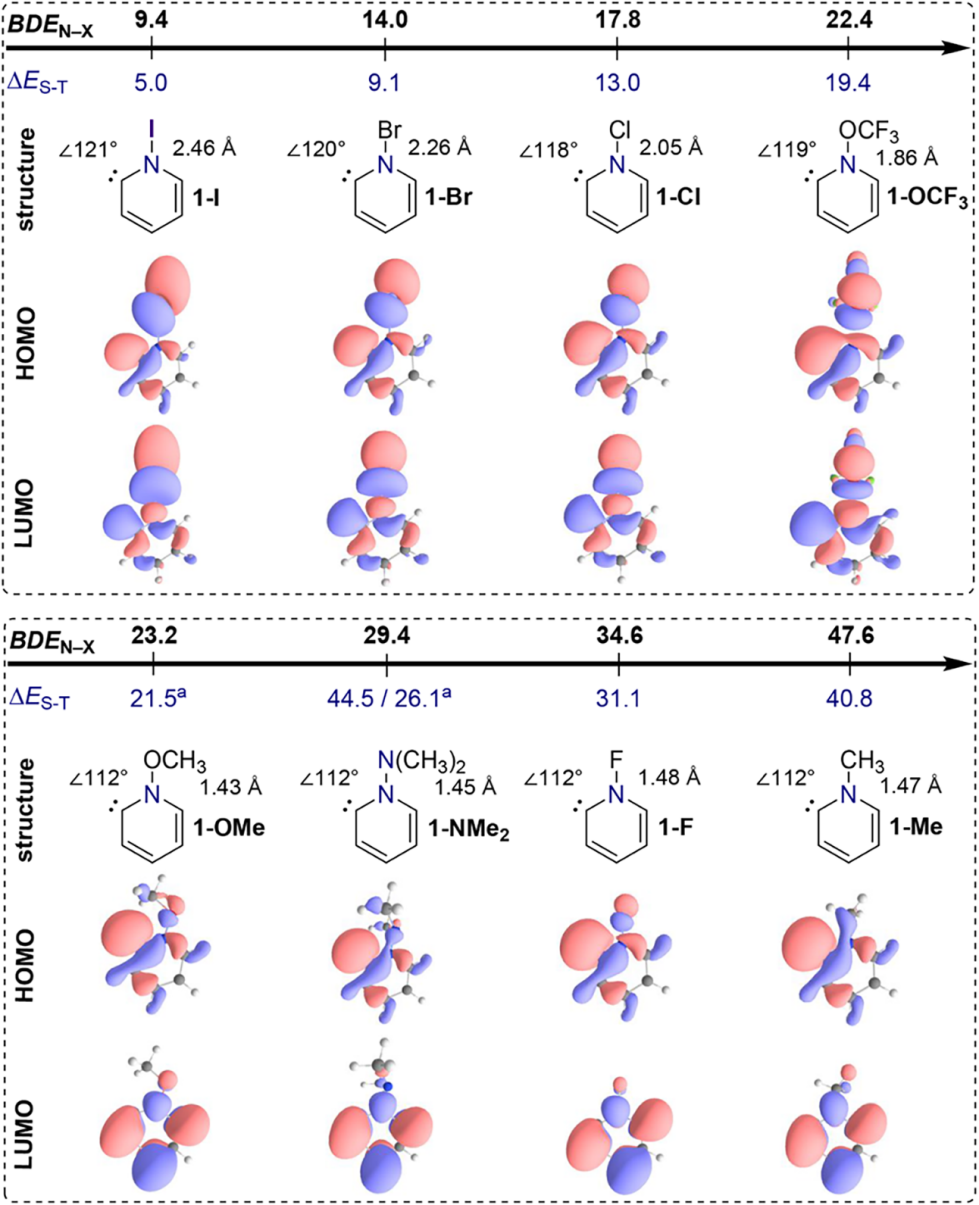
Structures and Kohn−Sham frontier orbitals of the investigated singlet carbenes. The bond dissociation energy of the N−X bond, *BDE*_N−X_, for fragmentation into radicals and the singlet−triplet energy gap, Δ*E*_S-T_, are given in kcal mol^−1^ and were computed at the DLPNO−CCSD­(T)/def2-TZVPP//B3LYP-D3/def2-TZVPP level of theory (^a^dissociated triplet).

The bond angles at the carbene center vary depending on the substituent, with **1-I**, **1-Br**, **1-Cl**, and **1-OCF**_**3**_ exhibiting larger angles between 118° and 121°, while **1-OMe**, **1-NMe**_**2**_, **1-F**, and **1-Me** all display a bond angle of 112°. This widening of the bond angle in the former carbenes suggests a significant resonance interaction between the lone pair and the N−X bond of the carbene, leading to a weakening of the latter. In line with this reasoning, carbenes with larger bond angles display N−X bond lengths which significantly exceed the sum of the covalent bond radii by 0.35 Å (**1-Cl**) to 0.52 Å (**1-OCF**_**3**_).^50^ In contrast, carbenes with smaller bond angles show minimal or no elongation of the N−X bond, with deviations not exceeding 0.13 Å for **1-OMe** and **1-F**, and no significant elongation for **1-NMe**_**2**_ and **1-Me**. Consequently, all carbenes with an increased bond angle feature two mixed σ-type frontier orbitals with contributions from the lone pair and an N−X σ* antibonding interaction of the carbene (Figure 2, top part). Conversely, carbenes without significant angle widening retain the expected π-type lowest unoccupied molecular orbital (LUMO), while the highest occupied molecular orbital (HOMO) remains more localized at the carbene center. Thus, based on our density functional theory structure optimizations, carbenes **1-I**, **1-Br**, **1-Cl**, and **1-OCF**_**3**_ can be classified as σ^2^σ*^0^-carbenes, whereas **1-OMe**, **1-NMe**_**2**_, **1-F**, and **1-Me** exhibit characteristics typical of σ^2^π^0^-carbenes.

Notably, we identified a second isomer of carbene **1-OCF**_**3**_, which features a smaller carbene bond angle of 112° and a shorter N−O bond of 1.48 Å (Figure S1). This structural reorganization is accompanied by a shift in electron configuration, with the carbene assuming a σ^2^π^0^ character. Notably, among all investigated substituents, only the OCF_3_ group exhibited this dual electronic structure, reinforcing the key finding from Figure 2 that this system is positioned at the borderline between dominant σ^2^σ*^0^ and σ^2^π^0^ configurations.

We further examined the lowest triplet states of all carbenes to determine whether the preferred frontier orbitals observed in the singlet state would persist in the state of higher multiplicity. The optimized structures, along with the singly occupied restricted open-shell Kohn−Sham orbitals, are shown in Figure S2. For the σ^2^σ*^0^ ground state carbenes **1-I**, **1-Br**, **1-Cl**, and **1-OCF**_**3**_ (as depicted in the top part of Figure 2), the frontier orbitals retain their character in the triplet state, transitioning into singly occupied molecular orbitals (SOMOs). The partial population of the antibonding N−X σ* orbitals indicates a pronounced weakening of the N−X bond, which is reflected in the observed bond elongations ranging from 0.39 Å (**1-Br**) to 0.44 Å (**1-Cl**). Nevertheless, the relative energies of the triplet state (Δ*E*_S-T_) remain consistently lower by 3.0−4.9 kcal mol^−1^ than the corresponding bond dissociation energies (*BDE*_N−X_), suggesting the presence of residual partial bonding interactions.

For the σ^2^π^0^ carbenes in the lower part of Figure 2, the triplet states exhibit greater diversity compared to the carbenes with a σ^2^σ*^0^ ground state. Carbenes **1-Me** and **1-N­(Me)**_**2**_ feature one σ and one π singly occupied molecular orbital in the triplet state. This electronic structure is associated with a pyramidalization at the nitrogen atom and a minor decrease in the N−X bond length. Their singlet−triplet gaps are large and exceed 40 kcal mol^−1^, consistent with typical N-heterocyclic carbenes. For carbene **1-N­(Me)**_**2**_, the computed bond dissociation energy is lower than the singlet−triplet gap, suggesting that the triplet state is inherently unstable. Indeed, a fully dissociated triplet radical pair, where the N−N bond is completely broken, exhibits a significantly lower energy than the intact triplet state. A similar dissociation occurs for carbene **1-OMe** where the atoms are separated by 3.17 Å in the triplet state. Carbene **1-F**, however, behaves differently: while its singlet state adopts a σ^2^π^0^ configuration, the triplet corresponds to the σ^1^σ*^1^ state. This deviation underscores its unique electronic flexibility, which is also reflected in its structural characteristics and reactivity patterns (*vide infra*).

### 3.2 Hydrogen Activation Reaction

Having analyzed the electronic structures of the substituted pyridinylidenes, we next examined their hydrogen activation reactions with a focus on the competing σ* and π-approach mechanistic pathways. All relevant stationary points on the potential energy surface were optimized using density functional theory (DFT) at the B3LYP-D3/def2-TZVPP level to provide a suitable reference for a subsequent high-level theoretical treatment. Energies of the transition states, some of which are subject to pronounced multireference character, were computed at the NEVPT2-(12,11)/def2-TZVPP level. The hereby computed activation barriers, $\Delta H_{0K}^{‡}$ , for the σ* and π-approach addition pathways are listed in Table 1 and plotted in Figure 3. We further followed the intrinsic reaction coordinate for all transition states, which also helped to distinguish between possible pyridine **A** and pyridinium cation **B** reaction products (Scheme 2). Proton transfer to produce a pyridinium ion pair **B** only occurred in the case of carbenes **1-I** and **1-Br**. The hydrogenation reaction energies, Δ_r_ *H*_0K_, can reliably be described with DLPNO−CCSD­(T)/def2-QZVPP coupled cluster theory and are also presented in Table 1.

**Table 1:** Activation Barriers, $\Delta H_{0K}^{‡}$ , and Zero-Point Corrected Reaction Energies, Δ_r_ *H*_0K_, for the Competing σ* and π Approach Hydrogenation Reaction Pathways are Given in kcal mol^−1^ for All Considered Carbenes

	σ*-approach			π-approach	
carbene	$\Delta H_{0K}^{‡}$ ^a^	$\Delta_{r}H_{0K}$ ^b^	product	$\Delta H_{0K}^{‡b}$ ^a^	$\Delta_{r}H_{0K}$ ^b^
**1-I**	4.3	−78.5	**B−I**		−38.7
**1-Br**	7.9	−86.2	**B−I**		−38.4
**1-Cl**	10.2	−95.3	**A-I**	32.6	−40.4
**1-OCF**_**3**_	20.6	−112.2	**A-I**	35.3	−44.3
**1-OMe**	37.1	−89.7	**A-I**	29.3	−32.3
**1-NMe**_**2**_	44.5	−70.0	**A-I**	25.7	−32.0
**1-F**	19.3	−114.1	**A-I**	33.2	−34.5
**1-Me**	52.7	−59.4	**A-I**	21.1	−36.2

a NEVPT2­(12,11)/def2-TZVPP//B3LYP-D3/def2-TZVPP.

b DLPNO−CCSD­(T)/def2-QZVPP/B3LYP-D3/def2-TZVPP.

**Figure 3:**
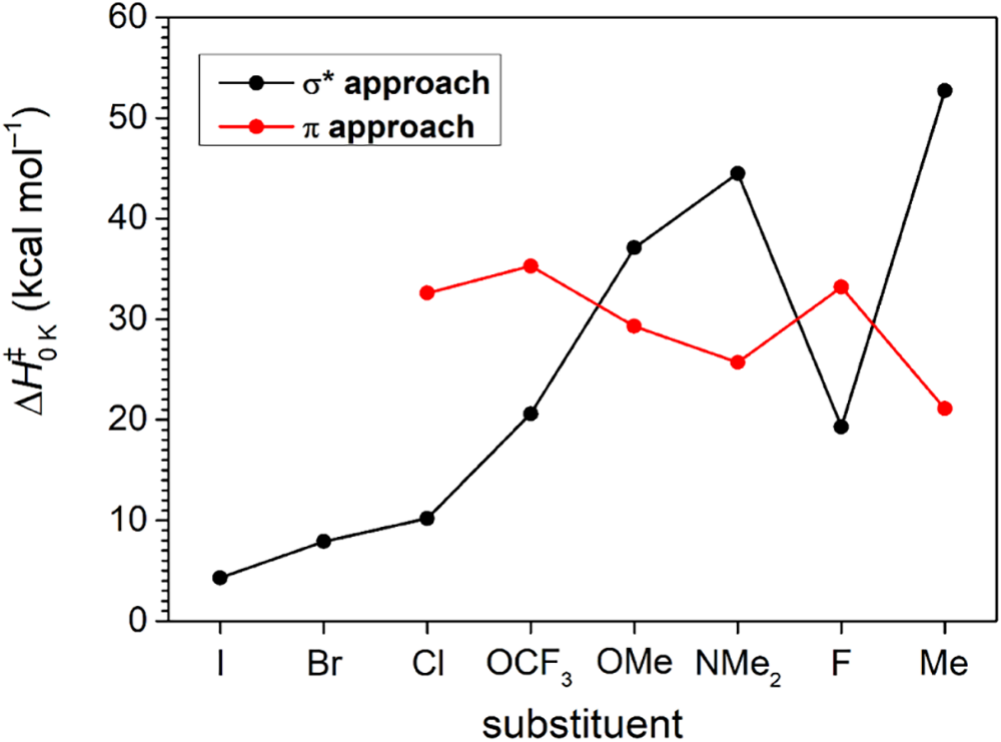
Plot of the activation barriers, $\Delta H_{0K}^{‡}$ , for the competing σ* and π approach hydrogenation reaction pathways depending on the substituent of the pyridinylidene. The substituents are ordered by the bond dissociation energy of the N−X bond, *BDE*_N−X_. The connecting lines are merely intended to guide the eye and have no physical meaning.

The trends in activation barriers, $\Delta H_{0K}^{‡}$ , for the competing σ*- and π-approach hydrogenation pathways as a function of the nitrogen substituent’s binding energy, *BDE*_N−X_, become apparent from Figure 3. Carbene **1-I** features the lowest σ*-approach reaction barrier of 4.2 kcal mol^−1^, in line with our previously reported experimental findings.^31^ Moving toward substituents with high bond dissociation energy, the activation barrier for the σ*-approach addition gradually increases, reaching 44.5 kcal mol^−1^ for carbene **1-NMe**_**2**_. However, this trend is interrupted by a significant drop to 19.3 kcal mol^−1^ for carbene **1-F**, before rising again to 52.7 kcal mol^−1^ for the methyl-substituted carbene **1-Me**. Thus, carbene **1-F** appears to be an outlier in the observed trend. The σ*-approach hydrogenation reaction energies show a large variation, ranging from −59.4 to −112.2 kcal mol^−1^.

The activation energies for the π-approach transition states exhibit less variation with the bond strengths of the substituents. Likewise, the hydrogenation reaction energies show little variation (−32.0 to −44.3 kcal mol^−1^), pointing toward an electronic stabilization of comparable magnitude for all pyridinylidenes.^51^ For carbenes **1-I** and **1-Br**, which feature the weakest N−X bonds, the π-approach transition structures could not be optimized, and in the case of **1-Br**, all optimization attempts converged back to the σ*-approach transition state. For **1-Cl** and **1-OCF**_**3**_, the π-approach activation barriers amount to 32.6 and 35.3 kcal mol^−1^, respectively. Beyond this point, the activation energies generally decrease with increasing bond strength, except for carbene **1-F**, which shows a slightly elevated barrier of 33.2 kcal mol^−1^. The lowest activation energy for the π-approach is observed for the methyl-substituted carbene **1-Me** at 21.1 kcal mol^−1^. Overall, carbenes with a σ^2^σ*^0^ electron configuration (**1-I**, **1-Br**, **1-Cl**, **1-OCF**_**3**_) favor the σ*-approach pathway with activation barriers that increase alongside the N−X bond dissociation energies. In contrast, carbenes with a σ^2^π^0^ electron configuration **(1-OMe**, **1-NMe**_**2**_−, and **1-Me**) show a preference for the π-approach pathway. However, the fluorine-substituted carbene (**1-F**) presents an apparent contradiction, as its DFT-optimized structure suggests a σ^2^π^0^ ground state, yet its hydrogenation barriers indicate a σ^2^σ*^0^-like reactivity. This apparent anomaly underscores the need for further investigations into the unique electronic structure of carbene **1-F**.

### 3.3 Dichotomy of the Fluorine-Substituted Carbene

Given that the fluorine-substituted carbene **1-F** prefers a σ*-approach reaction pathway, we surmised that it might be able to switch its preferred electron configuration from σ^2^π^0^ to σ^2^σ*^0^ at a negligible energetic cost. In order to substantiate our assumption, we elongated the N−F bond length in steps of 0.1 Å while simultaneously optimizing all other coordinates with the broken-symmetry BS-UB3LYP-D3/def2-TZVPP method. We further computed NEVPT2/def2-TZVPP single-point energies on top of all structures to obtain reliable energies. The resulting potential energy curve in the range of 1.48 to 2.08 Å is displayed in Figure 4.

**Figure 4:**
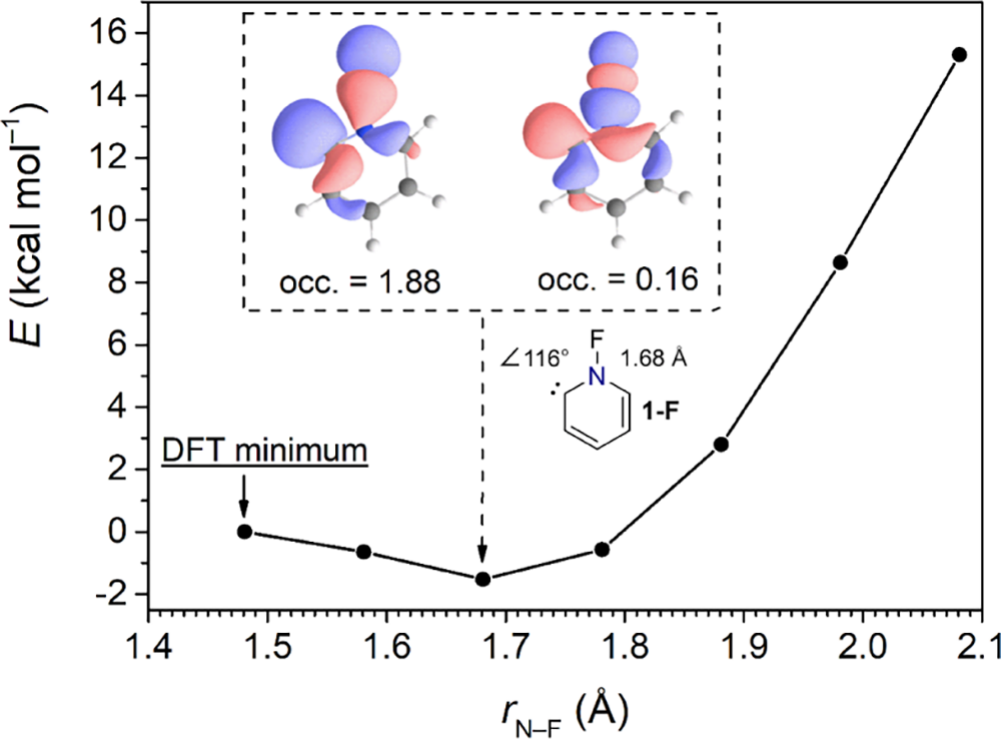
Electronic energy of carbene **1-F** upon stretching the N−F bond computed at the NEVPT2­(10,9)/def2-TZVPP/BS-UB3LYP-D3/def2-TZVPP level of theory. The electronic structure of the carbene changes from a σ^2^π^0^ to a dominant σ^2^σ*^0^ configuration without significant change of the electronic energy.

Consistent with our hypothesis, increasing the bond length from the density functional theory minimum leads to a decrease in the NEVPT2 energy, which reaches a minimum at 1.68 Å with a relative energy of −1.5 kcal mol^−1^. At this optimized structure, the carbene adopts a wider bond angle of 116°, and the carbene σ and N−F bond σ* natural orbitals show an extensive mixing and deviations from integral occupation numbers (inset Figure 4). This suggests that a fully relaxed, yet computationally demanding, NEVPT2 optimization would likely predict a σ^2^σ^*0^ ground state configuration for carbene **1-F**. However, the energetic cost of reverting to the σ^2^π^0^ configuration remains minimal, and the overall potential energy surface is relatively flat within the bond length range of 1.48−1.78, only increasing more steeply beyond this point. Consequently, carbene **1-F** appears highly adaptable, capable of interconverting between ground state configurations with minimal energy expenditure by adjusting the N−F bond length. This flexibility enables the σ*-approach hydrogenation pathway to remain energetically accessible, a behavior that starkly contrasts with other carbenes of similar N−X bond dissociation energy, such as **1-NMe**_**2**_ (Figure S3).

## 4 Conclusions

In summary, our computational analysis reveals a strong correlation between the preferred ground state electron configuration of the carbene and the bond dissociation energy of the substituent at nitrogen. Pyridinylidenes with weaker bonds tend to adopt a σ^2^σ*^0^ ground state, whereas those with substituents featuring a high bond dissociation energy typically display a σ^2^π^0^ singlet state. An exception is presented by the fluorine-substituted carbene, **1-F**, which can easily switch between configurations with slight variations in the N−F bond length, since the energy difference between these states is minimal. This trend in electronic structure extends to the hydrogenation reactivity of the carbenes: While σ^2^σ*^0^ carbenes prefer to react *via* the σ*-approach reaction pathway with activation energies increasing with the N−X bond strength, σ^2^π^0^ carbenes react through an out of plane π-approach reaction trajectory. Once more, the fluorine-substituted carbene **1-F** presents an exception, exhibiting a more flexible behavior favoring the σ*-approach reaction pathway.

## Supplementary Material



## Notes

Additional figures and tables; optimized geometries and energies (PDF)
