# Supplementary material for: Competing Modes of Hydrogen Activation in Singlet Pyridinylidenes: π-Approach vs σ*-Approach Reaction Pathways
Source: J Phys Chem A. 2025 Sep 1;129(36):8380–6. doi: 10.1021/acs.jpca.5c04771 (PMC12434657; doi:10.1021/acs.jpca.5c04771)
Supplement: Supplementary file 1 [file jp5c04771_si_001.pdf]

- Supporting Information -

Competing Modes of Hydrogen Activation in Singlet Pyridinylidenes: π-Approach vs. σ*-Approach Reaction Pathways

Gurli Schuster,[a] Virinder Bhagat,[a] and J. Philipp Wagner*[a,b]

[a]Institut für Organische Chemie, Eberhard Karls Universität Tübingen, Auf der Morgenstelle 18, 72076 Tübingen

[b]Institut für Organische und Analytische Chemie, Universität Bremen, Leobener Straße 7, 28359 Bremen

*Email: jpw@uni-bremen.de

Table of Contents

1. Additional Figures and Tables S2
2. Optimized Geometries and Energies S6

1. Additional Schemes and Figures

Table S1. T_1 diagnostic values of the substituted pyridinylidenes in their singlet states.

**Carbene**	**T_1 value**
**1-I**	0.0236
**1-Br**	0.0219
**1-Cl**	0.0257
**1-OCF$_3$** ($\sigma^2\pi^0$)	0.0148
**1-OCF$_3$** ($\sigma^2\sigma^{*0}$)	0.0189
**1-OMe**	0.0154
**1-NMe$_2$**	0.0133
**1-F**	0.0191
**1-Me**	0.0137

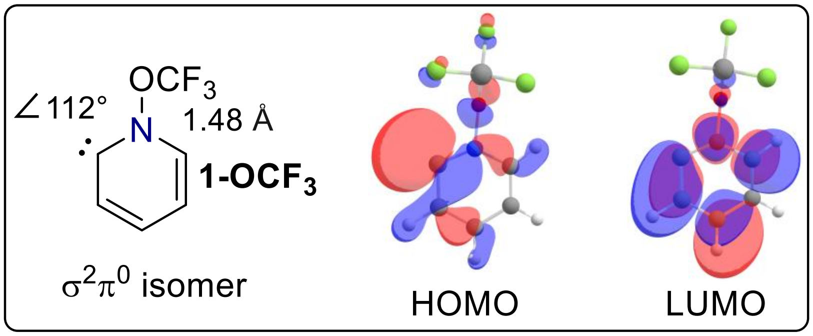

Figure S1. Structure and Kohn-Sham frontier orbitals of the $\sigma^2\pi^0$ isomer of carbene **1-OCF$_3$**.

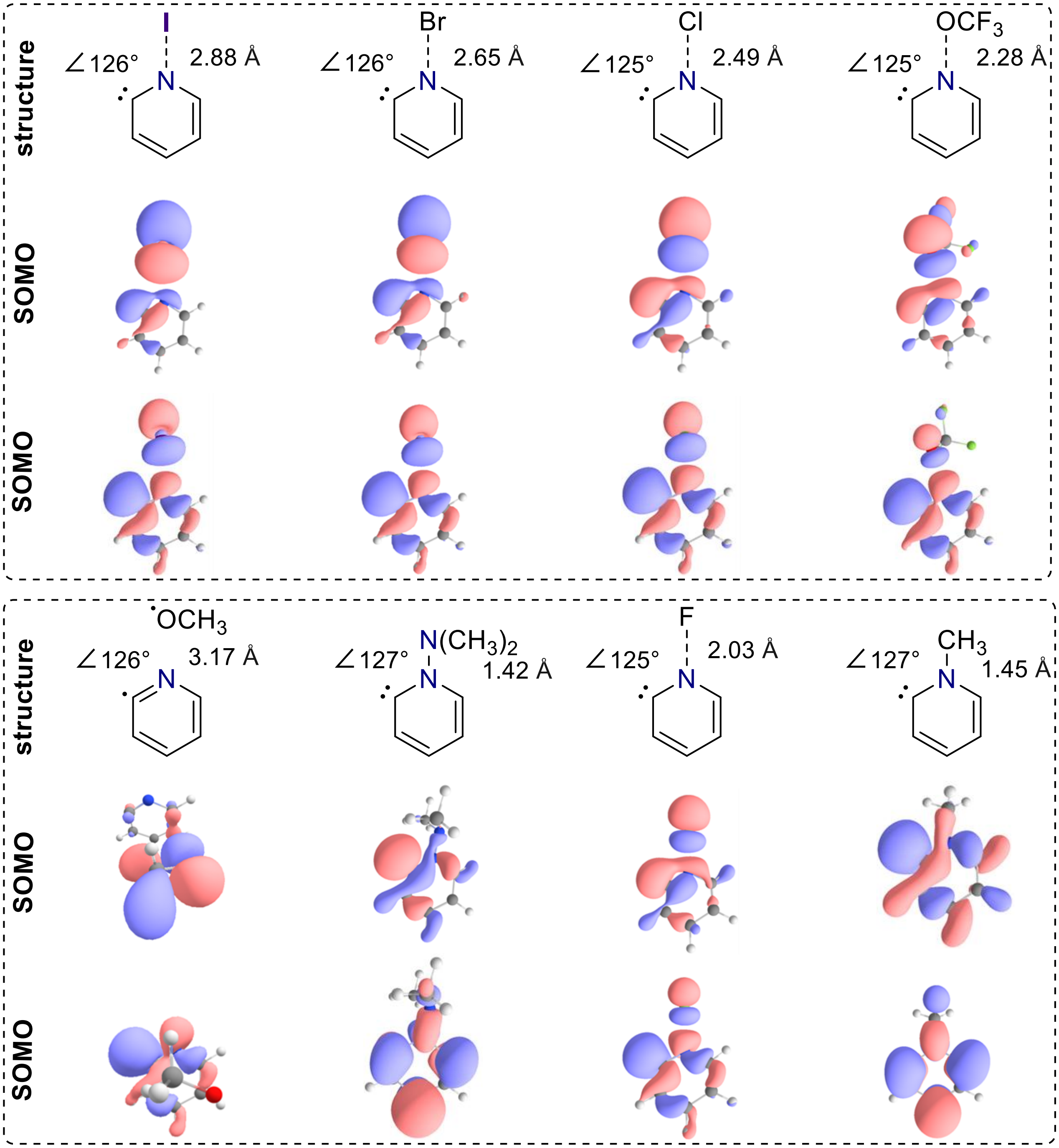

Figure S2. Structures and restricted open-shell Kohn-Sham singly occupied molecular orbitals of the investigated triplet carbenes.

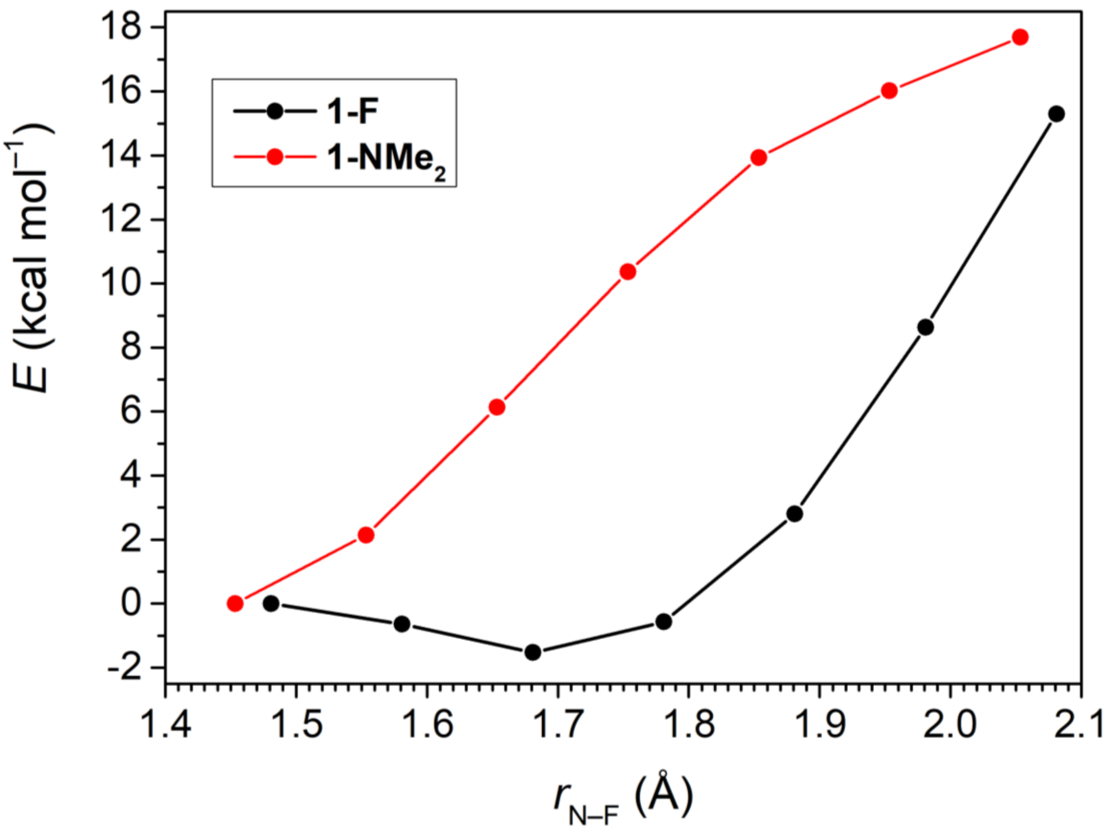

Figure S3. Electronic energy of carbene **1-NMe2** in comparison to **1-F** upon stretching the N−X bond computed at the NEVPT2(10,9)/def2-TZVPP/BS-UB3LYP-D3/def2-TZVPP level of theory.

2. Optimized Geometries and Energies

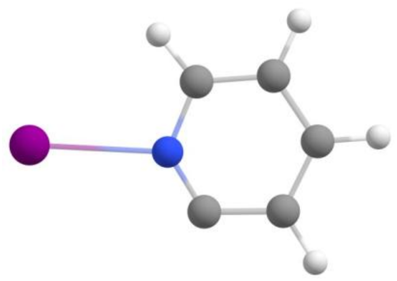

Figure S4. Optimized geometry of singlet **1-I** (in Å) at the B3LYP-D3/def2-TZVPP level of theory.

6	0.650978000	3.404075000	0.000000000
6	-0.677601000	3.017552000	0.000000000
6	-0.933516000	1.637499000	0.000000000
7	0.000000000	0.810414000	0.000000000
6	1.321772000	1.099108000	0.000000000
6	1.662641000	2.431590000	0.000000000
1	0.915136000	4.454890000	0.000000000
1	-1.480762000	3.739796000	0.000000000
1	2.027956000	0.283746000	0.000000000
1	2.707185000	2.707923000	0.000000000
53	-0.307833000	-1.630154000	0.000000000

ZPVE (kcal mol^{-1}): 48.38003
DLPNO-CCSD(T) (E_h): -544.49955
NEVPT2 (E_h): -544.43199

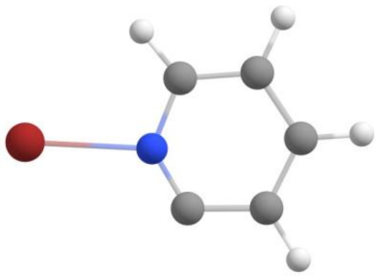

Figure S5. Optimized geometry of singlet **1-Br** (in Å) at the B3LYP-D3/def2-TZVPP level of theory.

6	1.227144000	2.706917000	0.000000000
6	-0.155074000	2.641674000	0.000000000
6	-0.723724000	1.357540000	0.000000000
7	0.000000000	0.350176000	0.000000000
6	1.349261000	0.307756000	0.000000000
6	1.987698000	1.526553000	0.000000000
1	1.728199000	3.667413000	0.000000000
1	-0.768352000	3.530686000	0.000000000
1	1.839003000	-0.652552000	0.000000000
1	3.067713000	1.554799000	0.000000000
35	-0.799383000	-1.765549000	0.000000000

ZPVE (kcal mol^{-1}): 48.53857
DLPNO-CCSD(T) (E_h): -2820.2497
NEVPT2 (E_h): -2819.9401

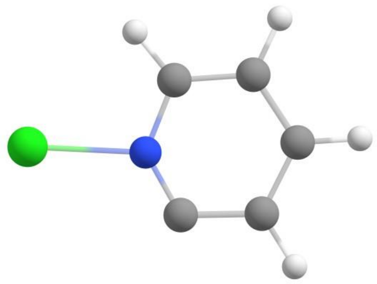

Figure S6. Optimized geometry of singlet **1-Cl** (in Å) at the B3LYP-D3/def2-TZVPP level of theory.

6	0.851776000	-2.142464000	0.000000000
6	1.753619000	-1.095170000	0.000000000
6	1.237215000	0.215384000	0.000000000
7	0.000000000	0.364409000	0.000000000
6	-0.966389000	-0.577837000	0.000000000
6	-0.528380000	-1.880418000	0.000000000
1	1.202035000	-3.167485000	0.000000000
1	2.820065000	-1.269076000	0.000000000
1	-1.997830000	-0.265551000	0.000000000
1	-1.254248000	-2.680439000	0.000000000
17	-0.873945000	2.218513000	0.000000000

ZPVE (kcal mol^{-1}): 48.65728
DLPNO-CCSD(T) (E_h): -706.94351
NEVPT2 (E_h): -707.03068

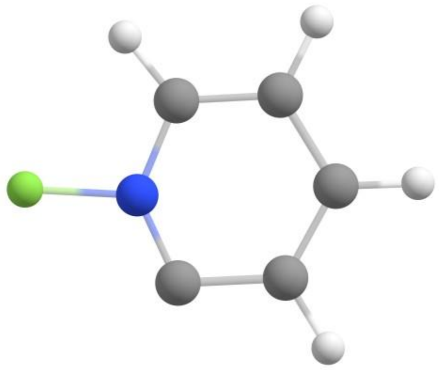

Figure S7. Optimized geometry of singlet **1-F** (in Å) at the B3LYP-D3/def2-TZVPP level of theory.

6	0.256972000	-1.796560000	0.000000000
6	1.359008000	-0.966882000	0.000000000
6	1.221968000	0.446467000	0.000000000
7	0.000000000	0.812185000	0.000000000
6	-1.159044000	0.122176000	0.000000000
6	-1.035051000	-1.242397000	0.000000000
1	0.375606000	-2.872909000	0.000000000
1	2.355041000	-1.389453000	0.000000000
1	-2.085299000	0.673555000	0.000000000
1	-1.921733000	-1.858321000	0.000000000
9	-0.287415000	2.265001000	0.000000000

ZPVE (kcal mol^{-1}):	49.21107
DLPNO-CCSD(T) (E_h):	-346.92665
NEVPT2 (E_h):	-346.87862

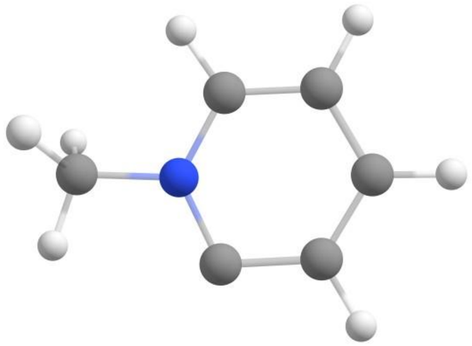

Figure S8. Optimized geometry of singlet **1-Me** (in Å) at the B3LYP-D3/def2-TZVPP level of theory.

6	-0.177400000	-1.857457000	0.000000000
6	-1.302029000	-1.062209000	0.000000000
6	-1.274004000	0.358910000	0.000000000
7	0.000000000	0.851621000	0.000000000
6	1.145371000	0.111903000	0.000000000
6	1.088513000	-1.252571000	0.000000000
1	-0.255272000	-2.938632000	0.000000000
1	-2.277248000	-1.534414000	0.000000000
1	2.081134000	0.654437000	0.000000000
1	2.000498000	-1.831076000	0.000000000
6	0.163360000	2.314459000	0.000000000
1	0.709312000	2.632117000	0.888957000
1	-0.830608000	2.745904000	0.000000000
1	0.709312000	2.632117000	-0.888957000

ZPVE (kcal mol^{-1}): 72.57345
DLPNO-CCSD(T) (E_h): -287.07678
NEVPT2 (E_h): -287.02607

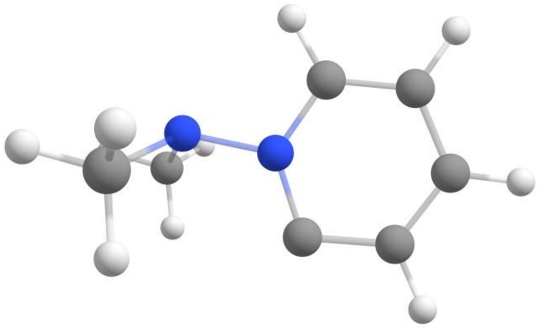

Figure S9. Optimized geometry of singlet **1-NMe₂** (in Å) at the B3LYP-D3/def2-TZVPP level of theory.

6	0.497314000	-2.505950000	0.000000000
6	1.476535000	-1.533671000	0.000000000
6	1.201772000	-0.143606000	0.000000000
7	-0.122776000	0.113891000	0.000000000
6	-1.139416000	-0.787210000	0.000000000
6	-0.851647000	-2.124611000	0.000000000
1	0.757156000	-3.558260000	0.000000000
1	2.516627000	-1.837656000	0.000000000
1	-2.136978000	-0.375672000	0.000000000
1	-1.652903000	-2.848570000	0.000000000
7	-0.588623000	1.490669000	0.000000000
6	-0.122776000	2.161640000	1.213729000
6	-0.122776000	2.161640000	-1.213729000
1	0.969610000	2.207041000	1.270348000
1	-0.534357000	3.170166000	1.213395000
1	-0.504323000	1.632216000	2.086119000
1	-0.504323000	1.632216000	-2.086119000
1	-0.534357000	3.170166000	-1.213395000
1	0.969610000	2.207041000	-1.270348000

ZPVE (kcal mol^{-1}): 100.53859
DLPNO-CCSD(T) (E_h): -381.57994
NEVPT2 (E_h): -381.50926

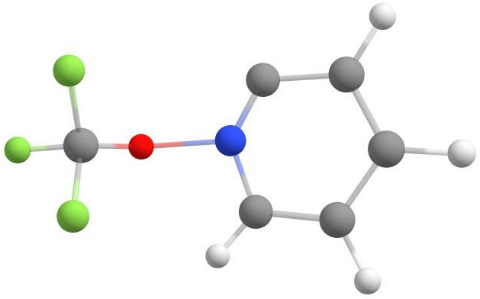

Figure S10. Optimized geometry of singlet $\sigma^2\sigma^{*0}$ **1-OCF$_3$** (in Å) at the B3LYP-D3/def2-TZVPP level of theory.

6	3.188555000	0.021304000	-0.265541000
6	2.597846000	-1.218538000	-0.080061000
6	1.243556000	-1.192628000	0.293110000
7	0.674158000	-0.119720000	0.410992000
6	1.119788000	1.135098000	0.264070000
6	2.449782000	1.206012000	-0.094349000
1	4.233165000	0.084144000	-0.544062000
1	3.141398000	-2.142599000	-0.204776000
1	0.446413000	1.962265000	0.411195000
1	2.904541000	2.174483000	-0.242350000
8	-1.070843000	-0.004905000	1.033445000
6	-1.869986000	-0.029492000	-0.000410000
9	-1.761552000	-1.150992000	-0.767085000
9	-3.160686000	0.055170000	0.391322000
9	-1.661664000	1.014539000	-0.875950000

ZPVE (kcal mol^{-1}): 59.2416
DLPNO-CCSD(T) (E_h): -659.68537
NEVPT2 (E_h): -659.6102

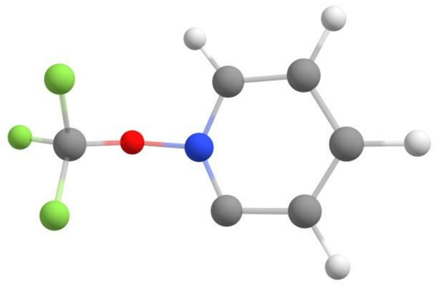

Figure S11. Optimized geometry of singlet $\sigma^2\pi^0$ **1-OCF$_3$** (in Å) at the B3LYP-D3/def2-TZVPP level of theory.

6	-3.082604000	-0.024548000	-0.280066000
6	-2.418377000	-1.213758000	-0.075925000
6	-1.056816000	-1.273369000	0.328090000
7	-0.537486000	-0.079749000	0.439318000
6	-1.088991000	1.151702000	0.270045000
6	-2.402109000	1.193078000	-0.102110000
1	-4.125602000	-0.014617000	-0.572057000
1	-2.944038000	-2.150053000	-0.211847000
1	-0.461122000	2.012142000	0.432974000
1	-2.885170000	2.147086000	-0.250223000
8	0.851564000	0.025034000	0.946829000
6	1.775559000	-0.022312000	-0.056929000
9	1.606644000	0.982315000	-0.945485000
9	2.966188000	0.122517000	0.521442000
9	1.761151000	-1.160537000	-0.747881000

ZPVE (kcal mol^{-1}): 59.86288
DLPNO-CCSD(T) (E_h): -659.69181
NEVPT2 (E_h): -659.59968

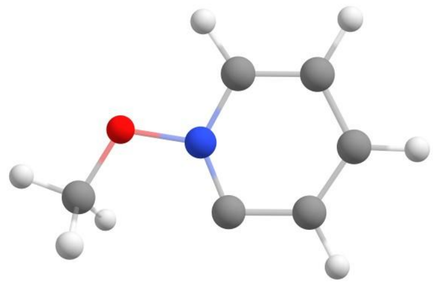

Figure S12. Optimized geometry of singlet **1-OMe** (in Å) at the B3LYP-D3/def2-TZVPP level of theory.

6	0.676401000	-2.157460000	0.000000000
6	-0.641799000	-1.753171000	0.000000000
6	-1.038317000	-0.391567000	0.000000000
7	0.000000000	0.426099000	0.000000000
6	1.324500000	0.130732000	0.000000000
6	1.689545000	-1.186608000	0.000000000
1	0.938352000	-3.208406000	0.000000000
1	-1.424384000	-2.501955000	0.000000000
1	2.011617000	0.962623000	0.000000000
1	2.736643000	-1.450324000	0.000000000
8	-0.178126000	1.844566000	0.000000000
6	-1.546350000	2.247892000	0.000000000
1	-2.072190000	1.892833000	0.885448000
1	-1.476724000	3.334261000	0.000000000
1	-2.072190000	1.892833000	-0.885448000

ZPVE (kcal mol^{-1}): 74.88017
DLPNO-CCSD(T) (E_h): -362.1731
NEVPT2 (E_h): -362.11171

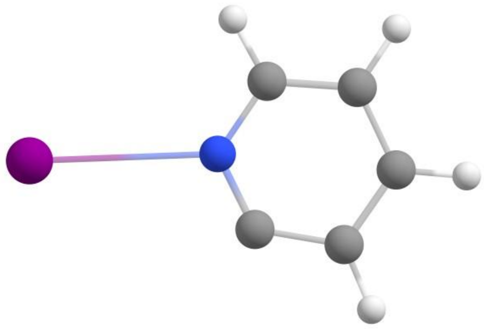

Figure S13. Optimized geometry of triplet **1-I** (in Å) at the UB3LYP-D3/def2-TZVPP level of theory.

6	0.652601000	3.444708000	0.000000000
6	-0.665487000	2.996750000	0.000000000
6	-0.822057000	1.621751000	0.000000000
7	0.128650000	0.751771000	0.000000000
6	1.406215000	1.168258000	0.000000000
6	1.699758000	2.521990000	0.000000000
1	0.862755000	4.506840000	0.000000000
1	-1.505772000	3.675009000	0.000000000
1	2.171660000	0.404037000	0.000000000
1	2.729473000	2.850278000	0.000000000
53	-0.771840000	-1.984953000	0.000000000

ZPVE (kcal mol^{-1}): 47.81701
DLPNO-CCSD(T) (E_h): -544.49074

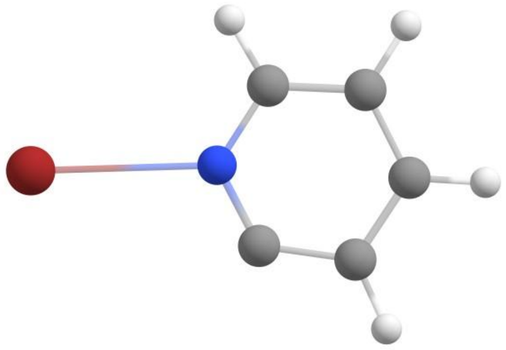

Figure S14. Optimized geometry of triplet **1-Br** (in Å) at the UB3LYP-D3/def2-TZVPP level of theory.

6	1.245067000	2.746145000	0.000000000
6	-0.143019000	2.633010000	0.000000000
6	-0.632142000	1.339605000	0.000000000
7	0.083012000	0.263829000	0.000000000
6	1.421551000	0.354884000	0.000000000
6	2.036008000	1.596832000	0.000000000
1	1.707124000	3.725237000	0.000000000
1	-0.792437000	3.495862000	0.000000000
1	1.974583000	-0.574548000	0.000000000
1	3.114628000	1.664147000	0.000000000
35	-1.261889000	-2.019589000	0.000000000

ZPVE (kcal mol^{-1}): 47.85669
DLPNO-CCSD(T) (E_h): -2820.2341

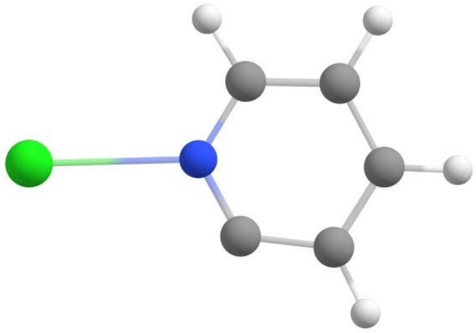

Figure S15. Optimized geometry of triplet **1-Cl** (in Å) at the UB3LYP-D3/def2-TZVPP level of theory.

6	0.867241000	-2.191453000	0.000000000
6	1.755239000	-1.118053000	0.000000000
6	1.172319000	0.135110000	0.000000000
7	-0.099892000	0.371499000	0.000000000
6	-0.968403000	-0.649015000	0.000000000
6	-0.507579000	-1.956174000	0.000000000
1	1.246419000	-3.205472000	0.000000000
1	2.826466000	-1.255370000	0.000000000
1	-2.019378000	-0.393918000	0.000000000
1	-1.212197000	-2.775563000	0.000000000
17	-0.816319000	2.758274000	0.000000000

ZPVE (kcal mol^{-1}): 47.90342
DLPNO-CCSD(T) (E_h): -706.92157

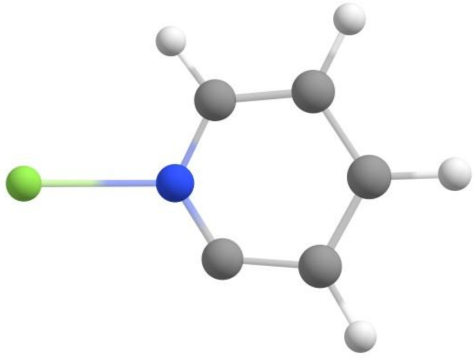

Figure S16. Optimized geometry of triplet **1-F** (in Å) at the UB3LYP-D3/def2-TZVPP level of theory.

6	0.256820000	-1.851252000	-0.030906000
6	1.385801000	-1.037170000	0.043214000
6	1.140256000	0.314295000	0.179894000
7	-0.038374000	0.860646000	0.224062000
6	-1.131659000	0.096600000	0.122097000
6	-1.014452000	-1.279766000	0.009087000
1	0.370389000	-2.924084000	-0.120038000
1	2.388420000	-1.437702000	0.011560000
1	-2.080718000	0.614631000	0.120693000
1	-1.902384000	-1.892062000	-0.056838000
9	-0.294043000	2.728727000	-0.536205000

ZPVE (kcal mol^{-1}): 48.10716
DLPNO-CCSD(T) (E_h): -346.87532

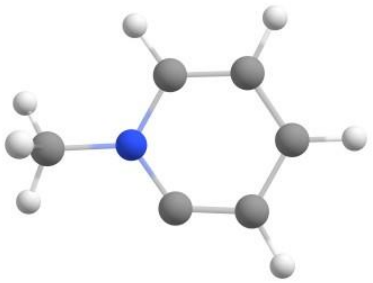

Figure S17. Optimized geometry of triplet **1-Me** (in Å) at the UB3LYP-D3/def2-TZVPP level of theory.

6	-0.171898000	-1.899089000	0.067659000
6	-1.338861000	-1.067967000	0.057722000
6	-1.155393000	0.256064000	-0.062582000
7	0.033207000	0.914130000	-0.213373000
6	1.190378000	0.099257000	-0.133973000
6	1.078484000	-1.255107000	-0.020710000
1	-0.256843000	-2.971742000	0.144588000
1	-2.332259000	-1.487934000	0.130625000
1	2.130952000	0.624701000	-0.195288000
1	1.990844000	-1.836051000	0.001768000
6	0.133643000	2.334394000	0.070288000
1	0.272604000	2.527900000	1.140484000
1	-0.773943000	2.833635000	-0.261715000
1	0.980025000	2.752918000	-0.473986000

ZPVE (kcal mol^{-1}): 70.56315
DLPNO-CCSD(T) (E_h): -287.00861

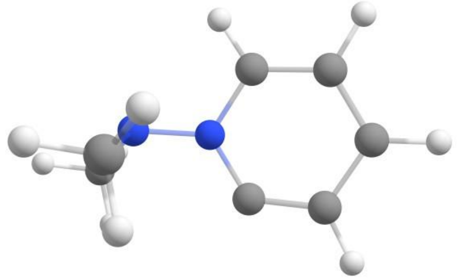

Figure S18. Optimized geometry of triplet **1-NMe$_2$** (in Å) at the UB3LYP-D3/def2-TZVPP level of theory.

6	0.516549000	-2.488001000	-0.251602000
6	1.503668000	-1.501984000	0.045313000
6	1.081589000	-0.265802000	0.359536000
7	-0.230134000	0.160569000	0.444546000
6	-1.200103000	-0.805814000	0.086901000
6	-0.835767000	-2.080281000	-0.224897000
1	0.799970000	-3.505192000	-0.473198000
1	2.557878000	-1.744842000	0.047959000
1	-2.212920000	-0.436484000	0.097350000
1	-1.621009000	-2.788195000	-0.453742000
7	-0.575602000	1.509077000	0.152041000
6	-0.068166000	2.393220000	1.192355000
6	-0.146557000	1.898540000	-1.191610000
1	1.030670000	2.414820000	1.233502000
1	-0.427218000	3.402668000	0.994655000
1	-0.449047000	2.066033000	2.157634000
1	-0.555746000	1.193933000	-1.913935000
1	-0.541702000	2.890170000	-1.408545000
1	0.947019000	1.919049000	-1.298070000

ZPVE (kcal mol^{-1}): 98.48073
DLPNO-CCSD(T) (E_h): -381.50576

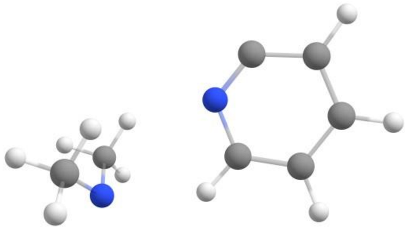

Figure S19. Optimized geometry of the dissociated triplet radical pair of **1-NMe$_2$** (in Å) at the UB3LYP-D3/def2-TZVPP level of theory.

6	0.459839000	-3.053988000	0.000000000
6	1.754977000	-2.546527000	0.000000000
6	1.846000000	-1.161296000	0.000000000
7	0.868826000	-0.334999000	0.000000000
6	-0.393320000	-0.810807000	0.000000000
6	-0.626394000	-2.176584000	0.000000000
1	0.296208000	-4.124734000	0.000000000
1	2.624443000	-3.187222000	0.000000000
1	-1.193347000	-0.079877000	0.000000000
1	-1.641097000	-2.549634000	0.000000000
7	-1.276649000	2.340892000	0.000000000
6	-0.550211000	2.676987000	1.192382000
6	-0.550211000	2.676987000	-1.192382000
1	0.358239000	2.060765000	1.260993000
1	-0.220107000	3.726111000	1.194716000
1	-1.160980000	2.494266000	2.076083000
1	-1.160980000	2.494266000	-2.076083000
1	-0.220107000	3.726111000	-1.194716000
1	0.358239000	2.060765000	-1.260993000

ZPVE (kcal mol^{-1}): 96.59712
DLPNO-CCSD(T) (E_h): -381.53204

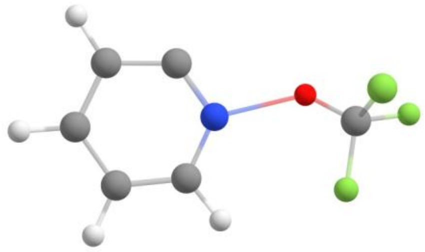

Figure S20. Optimized geometry of triplet **1-OCF$_3$** (in Å) at the UB3LYP-D3/def2-TZVPP level of theory.

6	-3.344944000	0.015020000	-0.191164000
6	-2.733185000	-1.228267000	-0.046464000
6	-1.356158000	-1.203880000	0.064701000
7	-0.622639000	-0.138173000	0.047175000
6	-1.193511000	1.067713000	-0.074610000
6	-2.569312000	1.174254000	-0.205066000
1	-4.420995000	0.078481000	-0.291557000
1	-3.296335000	-2.149634000	-0.027433000
1	-0.531039000	1.922175000	-0.065864000
1	-3.025302000	2.148050000	-0.312434000
8	1.365594000	-0.561092000	1.069216000
6	2.175208000	-0.013854000	0.171489000
9	1.931673000	1.305224000	-0.051966000
9	3.434629000	-0.113550000	0.648971000
9	2.145107000	-0.607535000	-1.038819000

ZPVE (kcal mol^{-1}): 58.31385
DLPNO-CCSD(T) (E_h): -659.65304

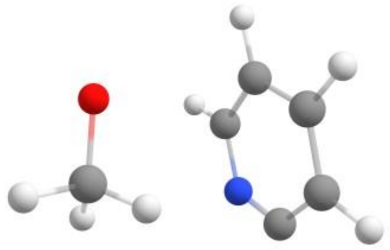

Figure S21. Optimized geometry of the triplet radical pair of carbene **1-OMe** (in Å) at the UB3LYP-D3/def2-TZVPP level of theory.

6	0.362347000	-1.500377000	0.607081000
6	-0.801025000	-1.775320000	-0.103650000
6	-0.915885000	-1.126389000	-1.327783000
7	-0.066244000	-0.320042000	-1.841653000
6	1.062525000	-0.041393000	-1.163655000
6	1.304785000	-0.620299000	0.073796000
1	0.531042000	-1.958213000	1.573730000
1	-1.559119000	-2.447287000	0.271108000
1	1.757486000	0.654028000	-1.614312000
1	2.206202000	-0.375193000	0.615672000
8	0.295609000	1.921225000	0.922117000
6	-1.020660000	1.915069000	0.551663000
1	-1.470126000	0.922588000	0.711615000
1	-1.556516000	2.581159000	1.251737000
1	-1.203445000	2.252790000	-0.474848000

ZPVE (kcal mol^{-1}): 71.20513
DLPNO-CCSD(T) (E_h): -362.13294

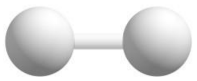

Figure S22. Optimized geometry of **H_2** (in Å) at the B3LYP-D3/def2-TZVPP level of theory.

1	0.000000000	0.000000000	0.371445000
1	0.000000000	0.000000000	-0.371445000

ZPVE (kcal mol^{-1}): 6.31818
NEVPT2 (E_h): -1.1659557

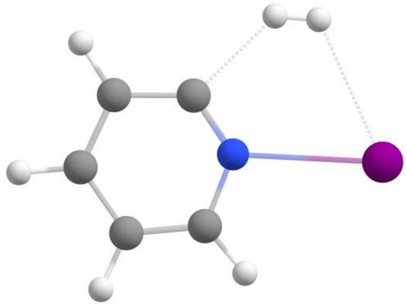

Figure S23. Optimized geometry of the σ*-approach transition state for **1-I** (in Å) at the B3LYP-D3/def2-TZVPP level of theory.

6	-3.558854000	0.100595000	0.000002000
6	-2.967091000	-1.155656000	0.000004000
6	-1.573467000	-1.140335000	0.000001000
7	-0.892479000	-0.108326000	-0.000003000
6	-1.389444000	1.141699000	-0.000005000
6	-2.765169000	1.256727000	-0.000002000
1	-4.638576000	0.189650000	0.000004000
1	-3.543357000	-2.068478000	0.000009000
1	-0.695053000	1.968538000	-0.000008000
1	-3.214438000	2.239403000	-0.000004000
53	1.724287000	0.041326000	0.000001000
1	-0.145169000	-2.522275000	-0.000021000
1	0.620888000	-2.457031000	-0.000029000

ZPVE (kcal mol^{-1}): 56.4502
NEVPT2 (E_h): -545.5939

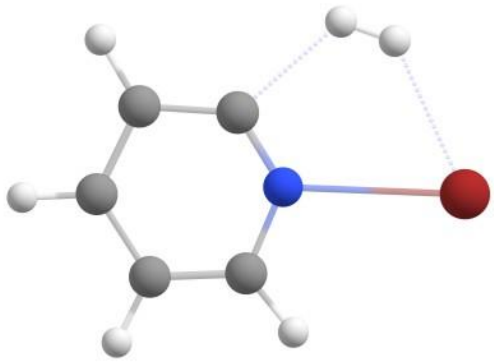

Figure S24. Optimized geometry of the σ*-approach transition state for **1-Br** (in Å) at the B3LYP-D3/def2-TZVPP level of theory.

6	3.112568000	-0.038109000	-0.000001000
6	2.477393000	1.200964000	-0.000001000
6	1.091237000	1.103865000	0.000001000
7	0.459340000	0.054561000	0.000001000
6	0.986442000	-1.174569000	0.000001000
6	2.369872000	-1.228601000	0.000000000
1	4.194788000	-0.083256000	-0.000001000
1	3.015238000	2.136350000	-0.000002000
1	0.317450000	-2.022425000	0.000001000
1	2.866058000	-2.188480000	0.000000000
35	-2.068717000	-0.051783000	0.000000000
1	-0.345587000	2.339073000	0.000017000
1	-1.083300000	2.067922000	-0.000012000

ZPVE (kcal mol^{-1}): 56.74444
NEVPT2 (E_h): -2821.0966

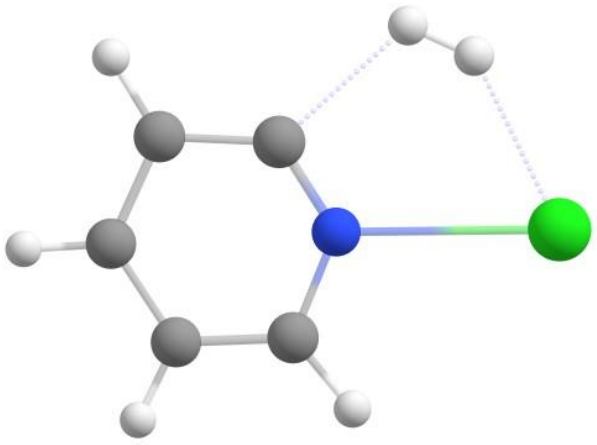

Figure S25. Optimized geometry of the σ*-approach transition state for **1-Cl** (in Å) at the B3LYP-D3/def2-TZVPP level of theory.

6	0.137494000	-2.456733000	0.000000000
6	1.321292000	-1.720686000	0.000000000
6	1.095173000	-0.351132000	0.000000000
7	0.000000000	0.183507000	0.000000000
6	-1.181410000	-0.435836000	0.000000000
6	-1.113818000	-1.820445000	0.000000000
1	0.185046000	-3.538744000	0.000000000
1	2.299208000	-2.176168000	0.000000000
1	-2.081485000	0.161050000	0.000000000
1	-2.025661000	-2.400264000	0.000000000
17	-0.229369000	2.607213000	0.000000000
1	2.196072000	1.192293000	0.000000000
1	1.773714000	1.863643000	0.000000000

ZPVE (kcal mol^{-1}): 56.89963
NEVPT2 (E_h): -708.18345

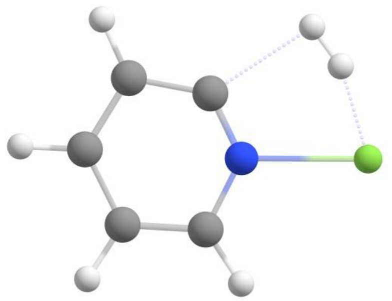

Figure S26. Optimized geometry of the σ*-approach transition state for **1-F** (in Å) at the B3LYP-D3/def2-TZVPP level of theory.

6	-0.153989000	-1.989110000	0.000000000
6	1.112113000	-1.398649000	0.000000000
6	1.016929000	-0.016750000	0.000000000
7	0.000000000	0.626937000	0.000000000
6	-1.247205000	0.169644000	0.000000000
6	-1.328890000	-1.217881000	0.000000000
1	-0.229830000	-3.069335000	0.000000000
1	2.032960000	-1.959737000	0.000000000
1	-2.074429000	0.863539000	0.000000000
1	-2.297358000	-1.697518000	0.000000000
9	0.237298000	2.728267000	0.000000000
1	2.316437000	1.542404000	0.000000000
1	1.722800000	2.094164000	0.000000000

ZPVE (kcal mol^{-1}): 57.06509
NEVPT2 (E_h): -348.01621

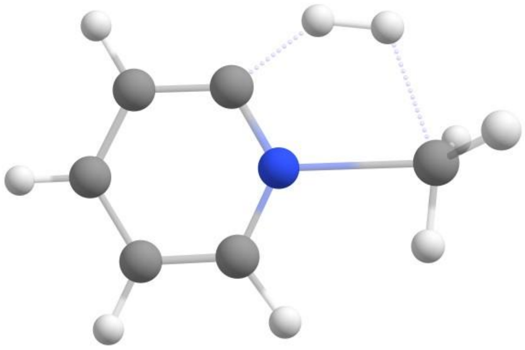

Figure S27. Optimized geometry of the σ*-approach transition state for **1-Me** (in Å) at the B3LYP-D3/def2-TZVPP level of theory.

6	2.027309000	0.082230000	0.000009000
6	1.265454000	1.242120000	0.000003000
6	-0.127836000	1.124529000	-0.000010000
7	-0.667398000	-0.042358000	-0.000020000
6	0.007125000	-1.190431000	-0.000011000
6	1.389160000	-1.162338000	0.000003000
1	3.109667000	0.132840000	0.000020000
1	1.740887000	2.214482000	0.000013000
1	-0.560921000	-2.114795000	-0.000020000
1	1.952313000	-2.084698000	0.000009000
1	-1.378868000	1.873372000	-0.000026000
1	-2.272696000	1.663348000	-0.000023000
6	-2.717351000	-0.166183000	0.000015000
1	-3.234036000	0.131923000	0.901980000
1	-3.234308000	0.132097000	-0.901740000
1	-2.513417000	-1.231631000	-0.000128000

ZPVE (kcal mol^{-1}): 79.13391
NEVPT2 (E_h): -288.10845

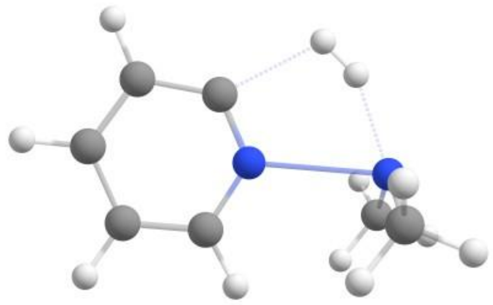

Figure S28. Optimized geometry of the σ*-approach transition state for **1-NMe₂** (in Å) at the B3LYP-D3/def2-TZVPP level of theory.

6	-2.813463000	-0.161905000	-0.000007000
6	-2.273369000	1.116852000	0.000002000
6	-0.878350000	1.182222000	0.000010000
7	-0.142796000	0.170781000	0.000008000
6	-0.600901000	-1.089171000	0.000004000
6	-1.970449000	-1.280741000	-0.000005000
1	-3.888254000	-0.299034000	-0.000015000
1	-2.895973000	1.999952000	0.000005000
1	0.103297000	-1.908308000	0.000010000
1	-2.374821000	-2.283136000	-0.000009000
1	0.622435000	2.465047000	0.000029000
1	1.301314000	2.041037000	0.000011000
6	2.404795000	-0.235648000	1.204175000
1	1.920784000	-1.225546000	1.225756000
1	3.483732000	-0.411650000	1.348419000
1	2.055538000	0.340526000	2.061520000
7	2.224744000	0.515192000	0.000001000
6	2.404787000	-0.235628000	-1.204187000
1	3.483723000	-0.411608000	-1.348454000
1	1.920789000	-1.225533000	-1.225776000
1	2.055505000	0.340555000	-2.061516000

ZPVE (kcal mol^{-1}): 106.26038
NEVPT2 (E_h): -382.60338

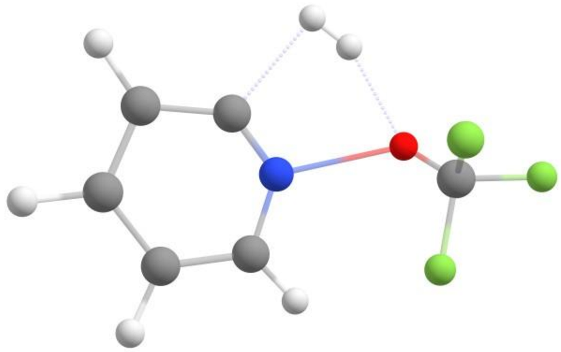

Figure S29. Optimized geometry of the σ*-approach transition state for **1-OCF$_3$** (in Å) at the B3LYP-D3/def2-TZVPP level of theory.

6	-3.329518000	-0.135970000	0.253128000
6	-2.806255000	1.159391000	0.163384000
6	-1.471823000	1.084801000	-0.168821000
7	-0.783639000	0.123712000	-0.364475000
6	-1.194993000	-1.148762000	-0.298694000
6	-2.540268000	-1.275217000	0.026165000
1	-4.375190000	-0.255314000	0.507860000
1	-3.373094000	2.059770000	0.331724000
1	-0.488963000	-1.944378000	-0.473628000
1	-2.973381000	-2.261835000	0.107970000
1	-0.187865000	2.679744000	-0.411985000
1	0.435020000	2.250011000	-0.577118000
6	2.010044000	0.037692000	-0.026394000
9	1.703884000	-1.337768000	0.111435000
9	3.376656000	0.061240000	-0.091005000
9	1.698704000	0.542520000	1.234265000
8	1.429078000	0.610060000	-0.989797000

ZPVE (kcal mol^{-1}): 67.06166
NEVPT2 (E_h): -660.74566

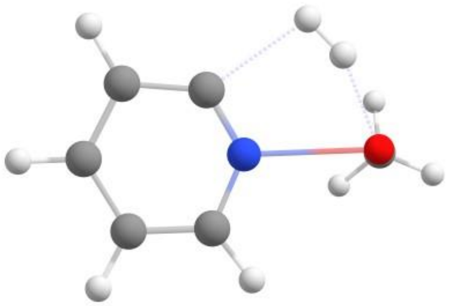

Figure S30. Optimized geometry of the σ*-approach transition state for **1-OMe** (in Å) at the B3LYP-D3/def2-TZVPP level of theory.

6	2.459045000	0.028543000	0.197344000
6	1.772298000	1.239430000	0.143297000
6	0.409245000	1.084797000	-0.090122000
7	-0.149562000	0.005916000	-0.232042000
6	0.422367000	-1.195926000	-0.200961000
6	1.790787000	-1.193824000	0.024060000
1	3.528233000	0.029872000	0.371044000
1	2.259728000	2.194238000	0.267790000
1	-0.187976000	-2.074842000	-0.352353000
1	2.333014000	-2.128032000	0.060919000
1	-1.240416000	2.160383000	-0.417965000
1	-1.764679000	1.558166000	-0.586976000
6	-2.867154000	-0.162682000	0.638918000
1	-2.277969000	-0.839897000	1.281794000
1	-3.001568000	0.777809000	1.197826000
1	-3.864207000	-0.618335000	0.527503000
8	-2.332094000	0.012149000	-0.625063000

ZPVE (kcal mol^{-1}): 81.18125
NEVPT2 (E_h): -363.21856

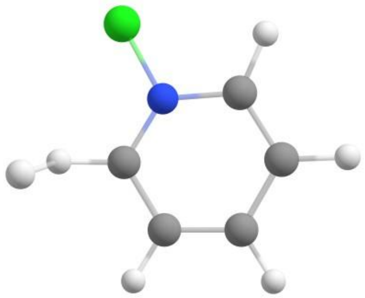

Figure S31. Optimized geometry of the π-approach transition state for **1-Cl** (in Å) at the B3LYP-D3/def2-TZVPP level of theory.

6	2.238555000	0.046383000	-0.195368000
6	1.567323000	-1.144187000	-0.153446000
6	0.167518000	-1.169869000	0.008592000
7	-0.454709000	0.009225000	0.122194000
6	0.183844000	1.216419000	0.227975000
6	1.531841000	1.253179000	0.062981000
1	3.301286000	0.076803000	-0.391258000
1	2.078436000	-2.094503000	-0.239513000
1	-0.430252000	2.075789000	0.448249000
1	2.048653000	2.196113000	0.162962000
17	-2.203191000	0.056281000	-0.150708000
1	-0.348702000	-2.107603000	0.408607000
1	-0.146703000	-2.379494000	1.613225000

ZPVE (kcal mol^{-1}): 56.30902
NEVPT2 (E_h): -708.14682

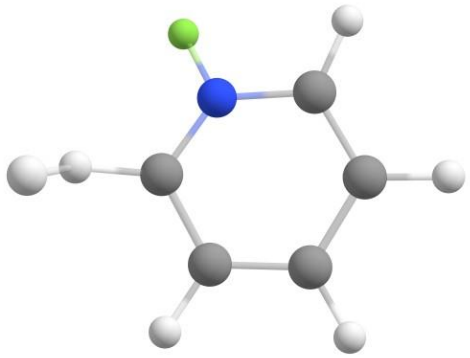

Figure S32. Optimized geometry of the π-approach transition state for **1-F** (in Å) at the B3LYP-D3/def2-TZVPP level of theory.

6	-1.824143000	-0.015577000	-0.171541000
6	-1.136222000	1.163510000	-0.137498000
6	0.270584000	1.162713000	0.010983000
7	0.843473000	-0.029115000	0.127808000
6	0.212855000	-1.236757000	0.208456000
6	-1.137341000	-1.243591000	0.068122000
1	-2.889062000	-0.026359000	-0.355652000
1	-1.631166000	2.122557000	-0.222072000
1	0.830657000	-2.097416000	0.413467000
1	-1.675637000	-2.173639000	0.174500000
9	2.202720000	-0.114244000	-0.298075000
1	0.816108000	2.099245000	0.371123000
1	0.505913000	2.325835000	1.535521000

ZPVE (kcal mol^{-1}): 57.08093
NEVPT2 (E_h): -347.99421

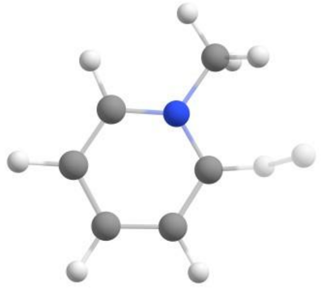

Figure S33. Optimized geometry of the π-approach transition state for **1-Me** (in Å) at the B3LYP-D3/def2-TZVPP level of theory.

6	1.908385000	0.107785000	-0.026865000
6	1.115054000	1.226824000	-0.087673000
6	-0.284383000	1.124027000	-0.152237000
7	-0.819743000	-0.132349000	-0.100485000
6	-0.060181000	-1.244513000	0.080337000
6	1.302342000	-1.161898000	0.092843000
1	2.986933000	0.190024000	-0.052081000
1	1.549085000	2.217842000	-0.093782000
1	-0.592246000	-2.178597000	0.187381000
1	1.890036000	-2.060590000	0.202409000
6	-2.274933000	-0.191198000	-0.083355000
1	-1.439059000	2.202993000	1.435757000
1	-0.996104000	1.945745000	0.295736000
1	-2.612043000	0.496080000	0.707581000
1	-2.674021000	0.151150000	-1.035661000
1	-2.612080000	-1.204371000	0.117756000

ZPVE (kcal mol^{-1}): 80.44858
NEVPT2 (E_h): -288.1609

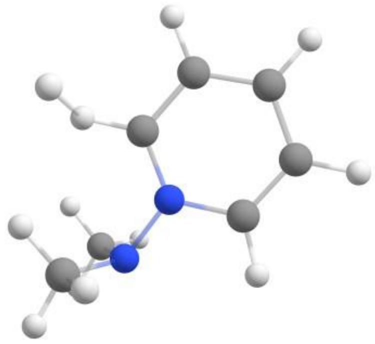

Figure S34. Optimized geometry of the π-approach transition state for **1-NMe₂** (in Å) at the B3LYP-D3/def2-TZVPP level of theory.

6	-2.560976000	0.147569000	0.133831000
6	-1.723585000	1.232042000	0.189831000
6	-0.331267000	1.079863000	0.116078000
7	0.147737000	-0.183841000	-0.047566000
6	-0.666444000	-1.263218000	-0.258604000
6	-2.016123000	-1.129652000	-0.150969000
1	-3.627998000	0.263353000	0.265138000
1	-2.104297000	2.241710000	0.272261000
1	-0.155575000	-2.185853000	-0.483486000
1	-2.648854000	-1.992277000	-0.296229000
7	1.542966000	-0.463474000	0.000766000
1	0.182486000	2.580264000	-1.384863000
1	0.284708000	1.983727000	-0.214597000
6	2.247733000	0.186646000	-1.107480000
1	2.222824000	1.280197000	-1.064402000
1	3.281667000	-0.154086000	-1.080930000
1	1.800050000	-0.127386000	-2.048357000
6	2.087686000	-0.121344000	1.313147000
1	1.536421000	-0.658645000	2.083176000
1	3.127025000	-0.444904000	1.338965000
1	2.044479000	0.953658000	1.525924000

ZPVE (kcal mol^{-1}): 108.54484
NEVPT2 (E_h): -382.63702

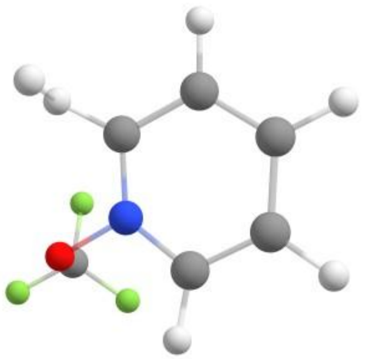

Figure S35. Optimized geometry of the π-approach transition state for **1-OCF$_3$** (in Å) at the B3LYP-D3/def2-TZVPP level of theory.

6	2.943884000	-0.130501000	-0.514694000
6	2.377095000	1.097133000	-0.323745000
6	1.081218000	1.228359000	0.230980000
7	0.459583000	0.054809000	0.491359000
6	1.057683000	-1.180921000	0.530988000
6	2.298202000	-1.294601000	-0.001938000
1	3.905557000	-0.223738000	-0.999614000
1	2.907415000	2.009641000	-0.563044000
1	0.482281000	-1.981078000	0.967777000
1	2.787843000	-2.256407000	-0.006722000
8	-0.875269000	0.113292000	0.929013000
6	-1.776067000	-0.009056000	-0.110261000
1	1.442541000	2.266296000	1.887494000
1	0.811450000	2.153008000	0.867760000
9	-1.640316000	0.951633000	-1.022826000
9	-2.979095000	0.066802000	0.440264000
9	-1.652156000	-1.187347000	-0.738910000

ZPVE (kcal mol^{-1}): 68.11877
NEVPT2 (E_h): -660.72398

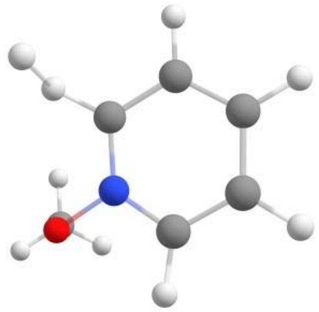

Figure S36. Optimized geometry of the π-approach transition state for **1-OMe** (in Å) at the B3LYP-D3/def2-TZVPP level of theory.

6	2.197658000	0.056119000	-0.365079000
6	1.463799000	1.198826000	-0.204426000
6	0.097277000	1.146077000	0.144698000
7	-0.425212000	-0.096188000	0.273057000
6	0.300487000	-1.252685000	0.293750000
6	1.615808000	-1.202728000	-0.044079000
1	3.228500000	0.103831000	-0.687534000
1	1.911794000	2.178543000	-0.306854000
1	-0.240714000	-2.143695000	0.571240000
1	2.200085000	-2.110244000	-0.046462000
8	-1.789918000	-0.220306000	0.507138000
6	-2.543701000	0.033963000	-0.688015000
1	-3.584781000	-0.048508000	-0.386485000
1	-2.342757000	1.038358000	-1.062809000
1	-2.312596000	-0.709290000	-1.454599000
1	0.043098000	2.239777000	1.880663000
1	-0.394770000	2.009560000	0.703246000

ZPVE (kcal mol^{-1}): 82.80282
NEVPT2 (E_h): -363.23351

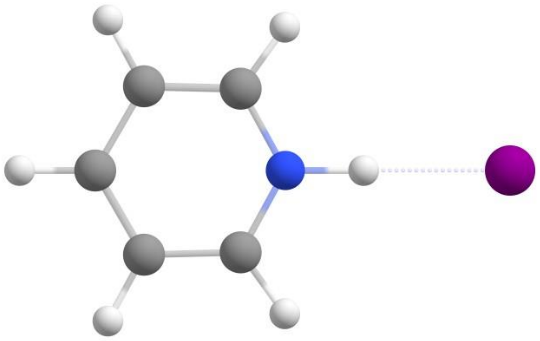

Figure S37. Optimized geometry of the σ*-approach reaction product for **1-I** (in Å) at the B3LYP-D3/def2-TZVPP level of theory.

6	3.910115000	0.000010000	-0.000250000
6	3.212537000	-1.204125000	-0.000227000
6	1.830365000	-1.170228000	-0.000138000
7	1.191059000	-0.000009000	-0.000076000
6	1.830348000	1.170217000	-0.000095000
6	3.212521000	1.204134000	-0.000183000
1	4.991528000	0.000016000	-0.000319000
1	3.728682000	-2.152688000	-0.000277000
1	1.198829000	2.048317000	-0.000039000
1	3.728651000	2.152706000	-0.000199000
53	-2.023209000	0.000000000	0.000129000
1	1.198856000	-2.048336000	-0.000113000
1	0.070815000	-0.000013000	-0.000004000

ZPVE (kcal mol^{-1}): 62.59746
DLPNO-CCSD(T) (E_h): -545.81109

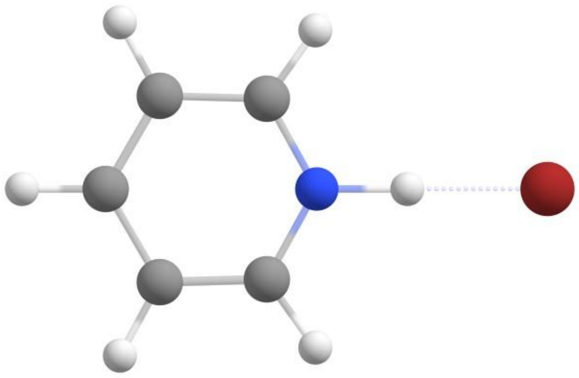

Figure S38. Optimized geometry of the σ*-approach reaction product for **1-Br** (in Å) at the B3LYP-D3/def2-TZVPP level of theory.

6	3.343355000	0.000013000	0.000006000
6	2.644663000	-1.202899000	0.000002000
6	1.261409000	-1.166169000	-0.000004000
7	0.616506000	-0.000002000	-0.000007000
6	1.261383000	1.166152000	-0.000005000
6	2.644652000	1.202904000	0.000002000
1	4.424833000	0.000005000	0.000010000
1	3.159964000	-2.152072000	0.000004000
1	0.635156000	2.048098000	-0.000007000
1	3.159945000	2.152083000	0.000004000
35	-2.363152000	0.000000000	0.000002000
1	0.635152000	-2.048091000	-0.000007000
1	-0.553040000	-0.000003000	-0.000015000

ZPVE (kcal mol^{-1}): 61.98466
DLPNO-CCSD(T) (E_h): -2821.5722

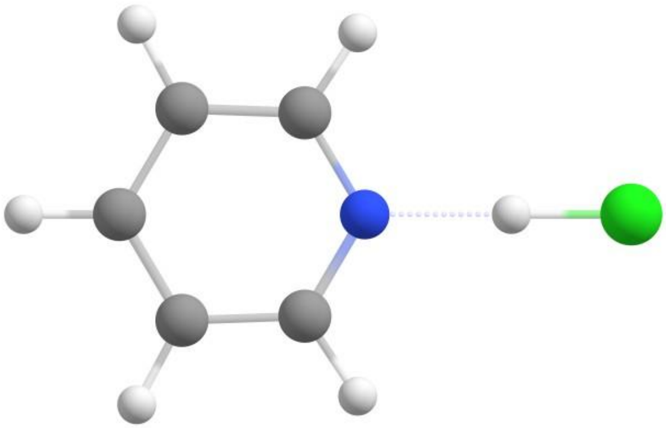

Figure S39. Optimized geometry of the σ*-approach reaction product for **1-Cl** (in Å) at the B3LYP-D3/def2-TZVPP level of theory.

6	-2.671932000	-0.000014000	-0.000001000
6	-1.966426000	-1.196923000	-0.000101000
6	-0.578881000	-1.148806000	-0.000098000
7	0.097024000	0.000015000	-0.000003000
6	-0.578904000	1.148819000	0.000092000
6	-1.966453000	1.196908000	0.000097000
1	-3.753736000	-0.000027000	0.000000000
1	-2.476562000	-2.149794000	-0.000179000
1	0.017755000	2.052808000	0.000166000
1	-2.476604000	2.149771000	0.000176000
17	3.106795000	0.000000000	0.000006000
1	0.017800000	-2.052780000	-0.000173000
1	1.752239000	0.000017000	0.000000000

ZPVE (kcal mol^{-1}): 61.42524
DLPNO-CCSD(T) (E_h): -708.27951

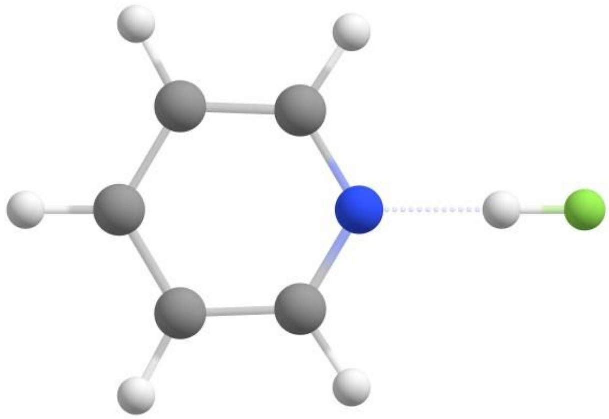

Figure S40. Optimized geometry of the σ*-approach reaction product for **1-F** (in Å) at the B3LYP-D3/def2-TZVPP level of theory.

6	-0.000013000	-2.126616000	0.000000000
6	-1.196077000	-1.420212000	0.000000000
6	-1.147615000	-0.032688000	0.000000000
7	0.000000000	0.647848000	0.000000000
6	1.147610000	-0.032699000	0.000000000
6	1.196059000	-1.420223000	0.000000000
1	-0.000017000	-3.208455000	0.000000000
1	-2.149429000	-1.929468000	0.000000000
1	2.053364000	0.560791000	0.000000000
1	2.149405000	-1.929488000	0.000000000
9	0.000028000	3.256794000	0.000000000
1	-2.053364000	0.560810000	0.000000000
1	0.000008000	2.294358000	0.000000000

ZPVE (kcal mol^{-1}): 63.72171
DLPNO-CCSD(T) (E_h): -348.2955

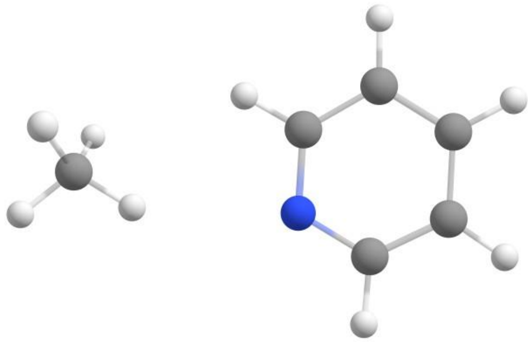

Figure S41. Optimized geometry of the σ*-approach reaction product for **1-Me** (in Å) at the B3LYP-D3/def2-TZVPP level of theory.

6	2.147388000	0.647118000	0.000019000
6	0.962155000	1.370901000	-0.000004000
6	-0.241527000	0.673751000	-0.000027000
7	-0.319882000	-0.657227000	-0.000029000
6	0.824245000	-1.341913000	-0.000007000
6	2.078264000	-0.740015000	0.000018000
1	3.104067000	1.152861000	0.000038000
1	0.964913000	2.452413000	-0.000004000
1	0.735320000	-2.422667000	-0.000009000
1	2.973549000	-1.346766000	0.000035000
1	-1.185585000	1.206966000	-0.000045000
1	-3.942851000	0.617309000	-0.888898000
6	-3.896504000	-0.010798000	0.000020000
1	-3.942813000	0.617298000	0.888947000
1	-4.744659000	-0.693769000	0.000034000
1	-2.966895000	-0.577319000	-0.000002000

ZPVE (kcal mol^{-1}): 84.27942
DLPNO-CCSD(T) (E_h): -288.35398

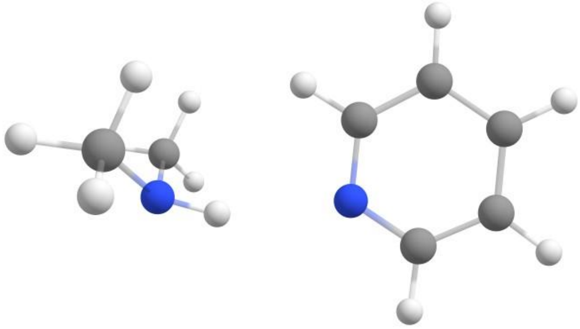

Figure S42. Optimized geometry of the σ*-approach reaction product for **1-NMe₂** (in Å) at the B3LYP-D3/def2-TZVPP level of theory.

6	3.019760000	0.609883000	0.000026000
6	2.897799000	-0.773720000	-0.000106000
6	1.622547000	-1.327876000	-0.000133000
7	0.507556000	-0.596576000	-0.000040000
6	0.634772000	0.731150000	0.000087000
6	1.864119000	1.380217000	0.000125000
1	3.995314000	1.077972000	0.000050000
1	3.769202000	-1.414013000	-0.000186000
1	-0.289221000	1.298057000	0.000161000
1	1.909141000	2.460584000	0.000230000
1	1.489526000	-2.403655000	-0.000234000
1	-1.688847000	-0.827692000	-0.000251000
6	-2.984103000	0.169527000	1.210952000
1	-2.522000000	1.172052000	1.262533000
1	-4.065343000	0.309346000	1.292565000
1	-2.655011000	-0.396710000	2.082660000
7	-2.667964000	-0.563061000	-0.000114000
6	-2.984336000	0.169994000	-1.210838000
1	-4.065584000	0.309932000	-1.292132000
1	-2.522164000	1.172499000	-1.262165000
1	-2.655510000	-0.395953000	-2.082834000

ZPVE (kcal mol^{-1}): 114.20293
DLPNO-CCSD(T) (E_h): -382.87706

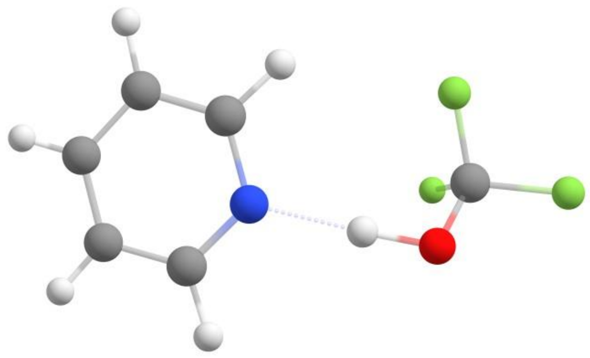

Figure S43. Optimized geometry of the σ*-approach reaction product for **1-OCF$_3$** (in Å) at the B3LYP-D3/def2-TZVPP level of theory.

6	3.623798000	0.365010000	0.187513000
6	3.258280000	-0.974467000	0.139135000
6	1.927137000	-1.290857000	-0.093259000
7	0.987187000	-0.360529000	-0.273339000
6	1.339609000	0.926731000	-0.225267000
6	2.646713000	1.334653000	0.002659000
1	4.652339000	0.647838000	0.367526000
1	3.985970000	-1.761177000	0.279112000
1	0.540691000	1.642641000	-0.370678000
1	2.886991000	2.387847000	0.034489000
1	1.595810000	-2.320887000	-0.137066000
1	-0.608894000	-0.725070000	-0.527057000
6	-2.318754000	-0.045619000	0.011877000
9	-1.988113000	1.259335000	-0.244655000
9	-3.618838000	-0.187719000	-0.270352000
9	-2.177118000	-0.187338000	1.358222000
8	-1.595912000	-0.899838000	-0.682229000

ZPVE (kcal mol^{-1}): 74.54035

DLPNO-CCSD(T) (E_h): -661.05244

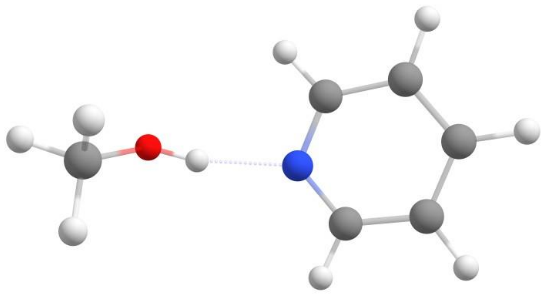

Figure S44. Optimized geometry of the σ*-approach reaction product for **1-OMe** (in Å) at the B3LYP-D3/def2-TZVPP level of theory.

6	-2.685564000	-0.207316000	0.198007000
6	-1.812834000	-1.282012000	0.086330000
6	-0.465158000	-1.022514000	-0.130664000
7	0.029432000	0.212197000	-0.236671000
6	-0.813672000	1.239624000	-0.129107000
6	-2.176985000	1.080651000	0.088139000
1	-3.741804000	-0.370079000	0.366901000
1	-2.164256000	-2.301441000	0.164340000
1	-0.379098000	2.227917000	-0.221632000
1	-2.819266000	1.946689000	0.167804000
1	0.252194000	-1.829139000	-0.226366000
1	1.901228000	0.119601000	-0.562218000
6	3.465026000	0.084146000	0.603709000
1	3.476634000	1.143532000	0.891015000
1	2.988549000	-0.481714000	1.414892000
1	4.499032000	-0.252494000	0.521907000
8	2.839486000	-0.130466000	-0.644803000

ZPVE (kcal mol^{-1}): 88.93977
DLPNO-CCSD(T) (E_h): -363.50225

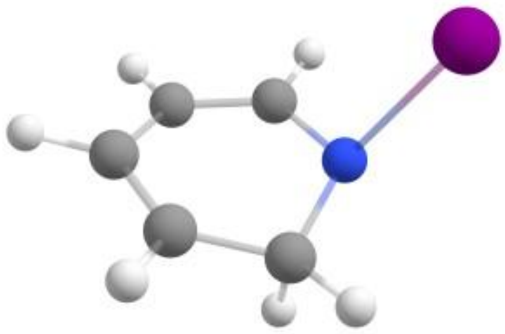

Figure S45. Optimized geometry of the π-approach reaction product for **1-I** (in Å) at the B3LYP-D3/def2-TZVPP level of theory.

6	2.741939000	-0.069003000	-0.786219000
6	2.165212000	-1.208425000	-0.366852000
6	1.302383000	-1.118239000	0.847931000
7	0.510820000	0.127244000	0.932141000
6	1.205558000	1.250517000	0.532194000
6	2.362571000	1.179991000	-0.175483000
1	3.430420000	-0.055933000	-1.621066000
1	2.319500000	-2.156534000	-0.863112000
1	0.737758000	2.194885000	0.781839000
1	2.921690000	2.082691000	-0.377111000
1	0.641401000	-1.970842000	0.971636000
1	1.941867000	-1.069094000	1.742144000
53	-1.400648000	-0.002357000	-0.140920000

ZPVE (kcal mol^{-1}): 62.6317
DLPNO-CCSD(T) (E_h): -545.7478

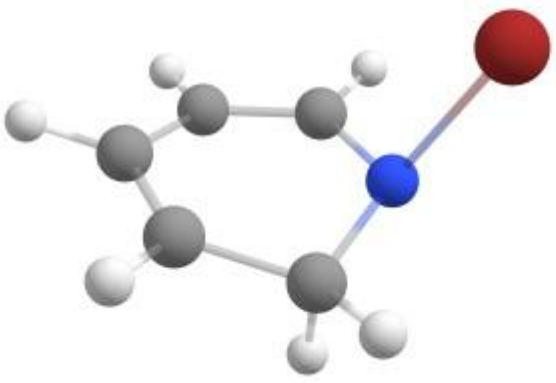

Figure S46. Optimized geometry of the σ*-approach reaction product for **1-Br** (in Å) at the B3LYP-D3/def2-TZVPP level of theory.

6	2.379754000	-0.052779000	-0.719338000
6	1.797549000	-1.199824000	-0.336667000
6	0.850853000	-1.132965000	0.816312000
7	0.060691000	0.111137000	0.887289000
6	0.764046000	1.250502000	0.525724000
6	1.946235000	1.192558000	-0.131220000
1	3.119044000	-0.028333000	-1.509080000
1	2.002533000	-2.144983000	-0.820299000
1	0.277360000	2.185005000	0.772355000
1	2.505255000	2.101671000	-0.303432000
1	0.174564000	-1.981133000	0.868711000
1	1.424203000	-1.117370000	1.754510000
35	-1.610240000	-0.003936000	-0.225790000

ZPVE (kcal mol^{-1}): 62.77397
DLPNO-CCSD(T) (E_h): -2821.4974

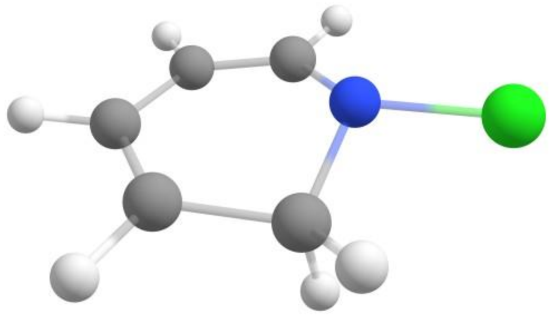

Figure S47. Optimized geometry of the σ*-approach reaction product for **1-Cl** (in Å) at the B3LYP-D3/def2-TZVPP level of theory.

6	2.254421000	0.033908000	-0.059891000
6	1.628286000	-1.147021000	-0.075136000
6	0.145422000	-1.224307000	0.196933000
7	-0.484048000	0.010576000	-0.287633000
6	0.155410000	1.212525000	0.013295000
6	1.495203000	1.259595000	0.116050000
1	3.326016000	0.091386000	-0.199473000
1	2.162104000	-2.078356000	-0.204518000
1	-0.475943000	2.086966000	0.048141000
1	1.987890000	2.210668000	0.246635000
17	-2.195369000	0.013542000	0.001623000
1	-0.315573000	-2.059720000	-0.327741000
1	-0.047333000	-1.363382000	1.275301000

ZPVE (kcal mol^{-1}): 63.22767
DLPNO-CCSD(T) (E_h): -708.1949

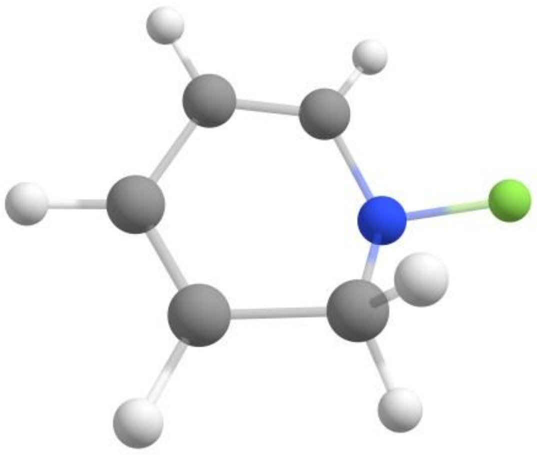

Figure S48. Optimized geometry of the σ*-approach reaction product for **1-F** (in Å) at the B3LYP-D3/def2-TZVPP level of theory.

6	1.853209000	0.030759000	0.036524000
6	1.225686000	-1.148685000	0.050231000
6	-0.270357000	-1.216368000	-0.157487000
7	-0.863009000	0.006992000	0.372823000
6	-0.248374000	1.205624000	0.004822000
6	1.087125000	1.260835000	-0.110772000
1	2.930069000	0.084840000	0.125658000
1	1.763122000	-2.084346000	0.113075000
1	-0.909022000	2.057556000	-0.041319000
1	1.578513000	2.210594000	-0.255825000
9	-2.220148000	0.029585000	-0.069606000
1	-0.505313000	-1.318258000	-1.230833000
1	-0.718707000	-2.058583000	0.366034000

ZPVE (kcal mol^{-1}): 63.86906
DLPNO-CCSD(T) (E_h): -348.16886

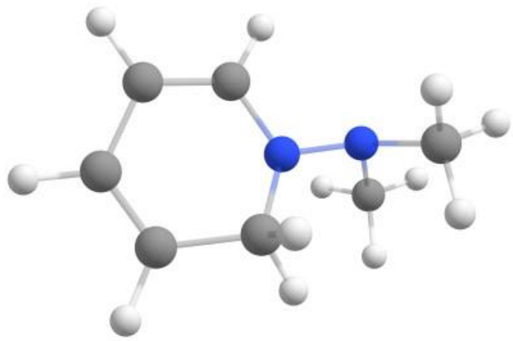

Figure S49. Optimized geometry of the σ*-approach reaction product for **1-NMe₂** (in Å) at the B3LYP-D3/def2-TZVPP level of theory.

6	-2.566133000	0.163167000	0.125228000
6	-1.798698000	1.252696000	-0.017669000
6	-0.352996000	1.108171000	-0.422251000
7	0.165405000	-0.163771000	0.091627000
6	-0.643833000	-1.269750000	0.060211000
6	-1.991308000	-1.162047000	0.033088000
1	-3.618284000	0.265715000	0.360660000
1	-2.198710000	2.251385000	0.093281000
1	-0.119128000	-2.211906000	0.126878000
1	-2.606722000	-2.047406000	0.053949000
7	1.552366000	-0.392721000	0.132270000
1	-0.248105000	1.157654000	-1.522163000
1	0.250387000	1.919601000	-0.012625000
6	2.212041000	-0.164216000	-1.149400000
1	2.243651000	0.894139000	-1.445676000
1	3.237443000	-0.526731000	-1.081248000
1	1.696731000	-0.728602000	-1.925248000
6	2.184784000	0.315082000	1.236803000
1	1.673222000	0.060367000	2.162361000
1	3.224123000	-0.006417000	1.308680000
1	2.177861000	1.409035000	1.117808000

ZPVE (kcal mol^{-1}): 114.74871
DLPNO-CCSD(T) (E_h): -382.81737

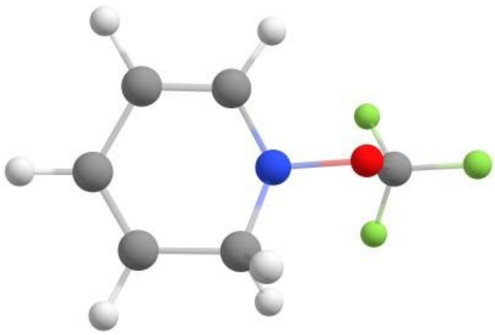

Figure S50. Optimized geometry of the σ*-approach reaction product for **1-OCF$_3$** (in Å) at the B3LYP-D3/def2-TZVPP level of theory.

6	-3.148001000	0.004984000	-0.318687000
6	-2.515319000	-1.162389000	-0.170100000
6	-1.127839000	-1.206925000	0.422452000
7	-0.434067000	0.036954000	0.079658000
6	-1.151730000	1.226816000	0.246888000
6	-2.473226000	1.249045000	0.022290000
1	-4.162496000	0.039236000	-0.693120000
1	-2.995372000	-2.105952000	-0.388358000
1	-0.550591000	2.097873000	0.455404000
1	-3.007690000	2.186089000	0.045493000
8	0.802169000	0.089852000	0.808130000
6	1.857997000	0.007752000	-0.051424000
1	-1.176671000	-1.326916000	1.518151000
1	-0.543506000	-2.032930000	0.022583000
9	1.851427000	-1.124623000	-0.771587000
9	2.958238000	0.031503000	0.698213000
9	1.902128000	1.031943000	-0.914550000

ZPVE (kcal mol^{-1}): 74.79945
DLPNO-CCSD(T) (E_h): -660.94468

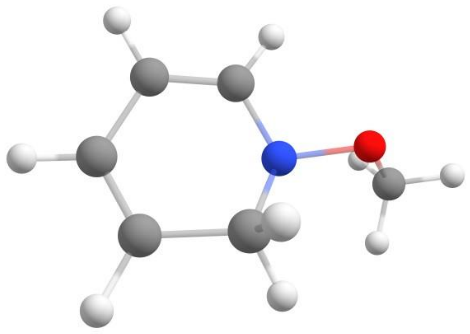

Figure S51. Optimized geometry of the σ*-approach reaction product for **1-OMe** (in Å) at the B3LYP-D3/def2-TZVPP level of theory.

6	-2.274630000	0.047493000	0.264690000
6	-1.598255000	1.195453000	0.141220000
6	-0.174101000	1.184373000	-0.361164000
7	0.458385000	-0.069502000	0.041623000
6	-0.278094000	-1.232067000	-0.124343000
6	-1.617805000	-1.221816000	0.002155000
1	-3.313711000	0.052552000	0.568208000
1	-2.067238000	2.154981000	0.310001000
1	0.311629000	-2.124455000	-0.272248000
1	-2.174003000	-2.145599000	-0.031268000
8	1.756077000	-0.171096000	-0.518041000
6	2.731978000	0.066982000	0.490770000
1	3.695972000	0.001568000	-0.011821000
1	2.617015000	1.062208000	0.928653000
1	2.673196000	-0.684420000	1.281471000
1	-0.141135000	1.290929000	-1.460590000
1	0.406414000	2.005015000	0.060598000

ZPVE (kcal mol^{-1}): 89.20477
DLPNO-CCSD(T) (E_h): -363.41129

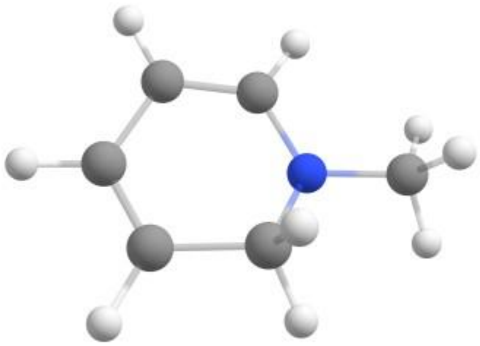

Figure S52. Optimized geometry of the σ*-approach reaction product for **1-Me** (in Å) at the B3LYP-D3/def2-TZVPP level of theory.

6	-1.898858000	0.011397000	-0.079043000
6	-1.251626000	-1.161221000	-0.094500000
6	0.222408000	-1.213561000	0.207907000
7	0.871746000	0.043922000	-0.165118000
6	0.177261000	1.210885000	0.027802000
6	-1.171606000	1.248859000	0.106744000
1	-2.967765000	0.047860000	-0.248733000
1	-1.767864000	-2.095914000	-0.265563000
1	0.775429000	2.112862000	0.042655000
1	-1.682654000	2.194164000	0.198224000
6	2.312151000	0.035675000	-0.065182000
1	0.399401000	-1.424505000	1.283512000
1	0.707333000	-2.023359000	-0.343634000
1	2.651127000	-0.167970000	0.961373000
1	2.726413000	-0.738516000	-0.713977000
1	2.717980000	0.995724000	-0.380397000

ZPVE (kcal mol^{-1}): 86.88675
DLPNO-CCSD(T) (E_h): -288.32107

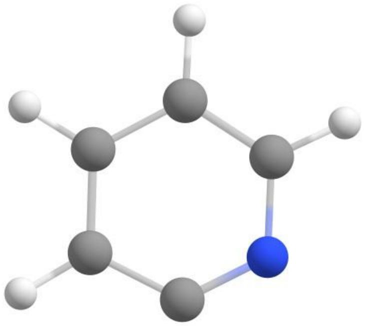

Figure S53. Optimized geometry of the 2-pyridyl radical (in Å) at the UB3LYP-D3/def2-TZVPP level of theory.

6	-1.012965000	0.869452000	0.000002000
6	-1.333725000	-0.483611000	-0.000002000
6	-0.247896000	-1.349798000	0.000005000
7	0.987718000	-1.019283000	-0.000003000
6	1.312841000	0.287310000	0.000004000
6	0.327486000	1.261269000	0.000000000
1	-1.798834000	1.614788000	0.000003000
1	-2.355978000	-0.832310000	-0.000011000
1	2.366096000	0.537331000	-0.000010000
1	0.600241000	2.307445000	-0.000005000

ZPVE (kcal mol^{-1}): 47.54702
DLPNO-CCSD(T) (E_h): -247.21809

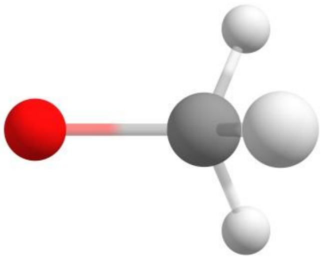

Figure S54. Optimized geometry of the methoxy radical (in Å) at the UB3LYP-D3/def2-TZVPP level of theory.

8	0.790395000	0.000743000	0.007213000
6	-0.572613000	-0.000556000	0.014226000
1	-0.873276000	-0.000834000	-1.052183000
1	-1.006287000	-0.909140000	0.454668000
1	-1.007915000	0.907368000	0.454453000

ZPVE (kcal mol^{-1}): 22.57991
DLPNO-CCSD(T) (E_h): -114.91043

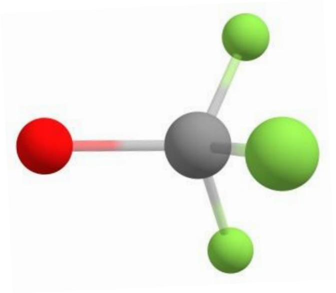

Figure S55. Optimized geometry of the trifluoromethoxy radical (in Å) at the UB3LYP-D3/def2-TZVPP level of theory.

8	0.757797000	0.000003000	1.167052000
6	0.005572000	0.000001000	0.040334000
9	-0.779375000	1.077635000	-0.045804000
9	0.881611000	-0.000115000	-0.972763000
9	-0.779548000	-1.077523000	-0.045702000

ZPVE (kcal mol^{-1}): 9.83834
DLPNO-CCSD(T) (E_h): -412.4287

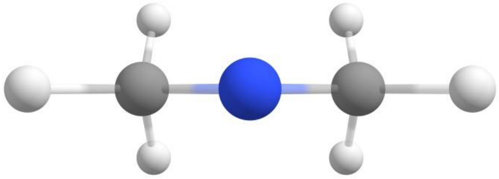

Figure S56. Optimized geometry of the NMe_2 radical (in Å) at the UB3LYP-D3/def2-TZVPP level of theory.

7	-0.000001000	-0.630644000	0.000000000
6	-0.000001000	0.169014000	1.192317000
6	-0.000001000	0.169014000	-1.192317000
1	0.880024000	0.829019000	1.231357000
1	-0.880026000	0.829016000	1.231368000
1	0.000009000	-0.464867000	2.078089000
1	0.000009000	-0.464867000	-2.078089000
1	-0.880026000	0.829016000	-1.231368000
1	0.880024000	0.829019000	-1.231357000

ZPVE (kcal mol^{-1}): 48.1945
DLPNO-CCSD(T) (E_h): -134.30727

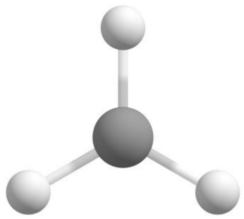

Figure S57. Optimized geometry of the methyl radical (in Å) at the UB3LYP-D3/def2-TZVPP level of theory.

6	0.000000000	0.000000000	0.000011000
1	0.539184000	-0.933895000	-0.000043000
1	0.539184000	0.933895000	-0.000043000
1	-1.078369000	0.000000000	-0.000043000

ZPVE (kcal mol^{-1}): 18.63378
DLPNO-CCSD(T) (E_h): -39.772577
